# SARS-CoV-2 membrane protein induces neurodegeneration via affecting Golgi-mitochondria interaction

**DOI:** 10.1186/s40035-024-00458-1

**Published:** 2024-12-27

**Authors:** Fang Wang, Hailong Han, Caifang Wang, Jingfei Wang, Yanni Peng, Ye Chen, Yaohui He, Zhouyang Deng, Fang Li, Yikang Rong, Danling Wang, Wen Liu, Hualan Chen, Zhuohua Zhang

**Affiliations:** 1 Department of Neurosciences, Hengyang Medical School, University of South China, Hengyang, 421009 China; 2 Institute of Molecular Precision Medicine and Hunan Provincial Key Laboratory of Molecular Precision Medicine, Xiangya Hospital, Central South University, Changsha, 410078 China; 3 Hunan Provincial Key Laboratory of Medical Genetics, College of Biological Sciences, Central South University, Changsha, 410078 China; 4 State Key Laboratory of Veterinary Biotechnology, Harbin Veterinary Research Institute, Chinese Academy of Agricultural Sciences, Harbin, 150001 China; 5 Fujian Provincial Key Laboratory of Innovative Drug Target Research, School of Pharmaceutical Sciences, Xiamen University, Xiamen, 361000 China

**Keywords:** COVID-19, Alzheimer’s disease, Mitochondria, PI4KIIIβ, Brain

## Abstract

**Background:**

Neurological complications are a significant concern of Coronavirus Disease 2019 (COVID-19). However, the pathogenic mechanism of neurological symptoms associated with severe acute respiratory syndrome coronavirus 2 (SARS-CoV-2) infection is poorly understood.

**Methods:**

We used *Drosophila* as a model to systematically analyze SARS-CoV-2 genes encoding structural and accessory proteins and identified the membrane protein (M) that disrupted mitochondrial functions in vivo. The M protein was stereotaxically injected to further assess its effects in the brains of wild-type (WT) and 5 × FAD mice. Omics technologies, including RNA sequencing and interactome analysis, were performed to explore the mechanisms of the effects of M protein both in vitro and in vivo.

**Results:**

Systematic analysis of SARS-CoV-2 structural and accessory proteins in *Drosophila* identified that the M protein induces mitochondrial fragmentation and dysfunction, leading to reduced ATP production, ROS overproduction, and eventually cell death in the indirect flight muscles. In WT mice, M caused hippocampal atrophy, neural apoptosis, glial activation, and mitochondrial damage. These changes were further aggravated in 5 × FAD mice. M was localized to the Golgi apparatus and genetically interacted with four wheel drive (FWD, a *Drosophila* homolog of mammalian PI4KIIIβ) to regulate Golgi functions in flies. *Fwd* RNAi, but not PI4KIIIα RNAi, reversed the M-induced Golgi abnormality, mitochondrial fragmentation, and ATP reduction. Inhibition of PI4KIIIβ activity suppressed the M-induced neuronal cell death. Therefore, M induced mitochondrial fragmentation and apoptosis likely through disruption of Golgi-derived PI(4)P-containing vesicles.

**Conclusions:**

M disturbs the distribution and function of Golgi, leading to mitochondrial abnormality and eventually neurodegeneration via a PI4KIIIβ-mediated mechanism. This study reveals a potential mechanism for COVID-19 neurological symptoms and opens a new avenue for development of therapeutic strategies targeting SARS-CoV-2 M or mitochondria.

**Supplementary Information:**

The online version contains supplementary material available at 10.1186/s40035-024-00458-1.

## Background

The Coronavirus Disease 2019 (COVID-19) pandemic caused by the severe acute respiratory syndrome coronavirus 2 (SARS-CoV-2) infection, presents a significant global health challenge [1–3]. Current SARS-CoV-2 vaccines are proven highly effective in combating COVID-19 [4–9]. However, a notable challenge arises from neurological complications that persist even after COVID-19 patient recovery [3, 10–12]. Approximately 60% of hospitalized COVID-19 patients exhibit neurological symptoms [13]. Older adults with COVID-19 are at a significantly increased risk of new diagnosis of AD [14]. Long-term cognitive decline is common after SARS-CoV-2 infection [15]. Autopsies have confirmed presence of coronaviruses in the central nervous system (CNS), particularly in the brain [16–19]. While SARS-CoV-2 invasion and transmission in the CNS remain to be understood, it is crucial to gain insights into its pathogenic mechanism within the CNS.

SARS-CoV-2 genome is a linear, positive-sense, single-stranded RNA of about 30 Kb [20]. The 5ʹ end of the genome harbors two large open-reading frames (orfs), orf1a and orf1b. These two orfs encode 15–16 non-structural proteins and 15 of them function in viral replication and transcription. The 3’ end of the genome encodes four structural proteins (S, E, M, and N proteins) and six accessory factors (ORF3a, ORF6, ORF7a, ORF7b, ORF8, and ORF10) [21]. Different studies have also reported variable numbers of ORFs for accessory proteins [21, 22]. The accessory factors participate in modulating host responses and determining viral pathogenicity [23, 24].

*Drosophila* is an important model for systematically probing functional interactions between viral and host proteins [25, 26]. Studies of the influenza virus [27] and the Zika virus [28–30] using *Drosophila* have led to insights into molecular functions of viral factors. *Drosophila* has also been employed to study a variety of pathogenic factors including proteins relevant to SARS-CoV-2 infection, such as ACE2-related receptors [31], ORF6a [32], and NSP6 [33]. In this study, we aimed to define pathogenic mechanisms of SARS-CoV-2 by systematically studying its genes encoding structural and accessory proteins. We first expressed three structural proteins, envelope protein (E protein, designated as E), membrane protein (M protein, designated as M) and nucleocapsid protein (N protein, N), and six accessory proteins (ORF3a, ORF6, ORF7a, ORF7b, ORF8, and ORF10) in *Drosophila,* identifying that SARS-CoV-2 M causes abnormalities of mitochondrial structures and function.

We further investigated the effects of M in the mouse brain, finding that it induced hippocampal atrophy and neural apoptosis, and these effects exacerbated in 5 × FAD mice. Additionally, M was localized to the Golgi apparatus and interacted genetically with FWD (PI4KIIIβ) to regulate Golgi distribution and function, thereby contributing to mitochondrial damage and neurodegeneration.

## Materials and methods

### Plasmids, *Drosophila* lines, mice, and virus

Plasmids were constructed as follows. cDNA fragments were PCR-amplified from human codon-optimized SARS-CoV-2 genes (*Orf3a*, *E*, *M*, *Orf6*, Orf*7a*, *Orf7*b, *Orf8, N*, and *Orf10*), respectively. The obtained DNA fragments were subcloned into the pUAST-attB vector (Unihuaii Company, Zhuhai, China) using EcoRI and Kpn1 restriction enzymes. All plasmids were sequence-confirmed. Common sequencing primers: forward sequencing primer (CMV): 5ʹ-CGCAAATGGGCGGTAGGCGTG-3ʹ, reverse sequencing primer (BGH): 5ʹ- TAGAAGGCACAGTCGAGG-3ʹ.

Flies carrying mhc-gal4 (Stock # 55133), elav-gal4 (Stock # 458), mef2-gal4 (Stock # 27390), uas-mito-GFP (Stock # 8443), uas-Golgi-GFP (Stock # 31422), uas-marf (Stock # 67157), uas-p35 (Stock # 5072), and uas-DIAP1 (Stock # 6657) were obtained from the Bloomington *Drosophila* Stock Center (BDSC, Bloomington, IN). Fly lines harboring uas-drp1 RNAi (Stock # CG3210), uas-fwd RNAi (Stock # CG7004), and uas-PI4KIIIα RNAi (Stock # CG10260) were obtained from the Tsinghua Fly Center (Beijing, China).

For experiments involving transgenic flies, the constructs were injected into the *w*^*1118*^ line (Stock # 3605, BDSC). Multiple independent fly lines for each construct were generated and characterized (Unihuaii). Briefly, constructs with the attB sequence were injected into flies (y1, w67c23; P(CaryP) attP2) to initiate the φC31 integrase-mediated site-specific integration (UniHuaii). The resulting flies (G0) were crossed to double balancer to produce the F1 generations. Fly strains were grown on standard cornmeal medium at 25 °C.

Age-matched wild-type (WT) and 5 × FAD mice (Jackson Laboratory, Bar Harbor, ME) were housed under a 12 h light/12 h dark cycle with free access to water and standard rodent chow diet. The following primers were used for genotyping 5 × FAD mice. PS1: Forward 5′-AATAGAGAACGGCAGGAGCA–3′, Reverse 5′-GCCATGAGGGCACTAATCAT–3′; Control: Forward 5′- CTAGGCCACAGAATTGAAAGATCT–3′, Reverse 5′- GTAGGTGGAAATTCTAGCATCATCC–3′. All animal experimental procedures were approved by the Institutional Animal Care and Ethics Review Committee for Animal Experimentation at Central South University.

To obtain the AAV2/9-hSynapsin-3xflag-M-CMV-EGFP-WPRE, human complementary DNA (cDNA) for 3xflag-M was excised from a pcDNA3.1-3xflag-M plasmid made in our laboratory using EcoRI and HindIII restriction enzymes and subcloned into the AAV2/9- hSynapsin-CMV-EGFP-WPRE vector (BrainVTA Company, Wuhan, China). The human synapsin promoter was used to restrict transgene expression only to neurons for all AAV2/9 viral vectors applied in this study. Additionally, the Woodchuck hepatitis virus post-transcriptional regulatory element (WPRE) was incorporated to enhance messenger RNA (mRNA) stability and sustain transgene expression. Throughout the paper, AAV viruses are referred to as AAV2/9 in which the first number refers to genotype and the second number refers to serotype. For the Aβ engulfment experiment by microglia, viruses carried a Flag-tag (BrainVTA Company) instead of GFP. Expression of M protein was validated by an anti-Flag tag antibody.

### Cell culture and transfection

HEK293 cells and Schneider’s *Drosophila* Line 2 (S2 cell line) were obtained from American Type Culture Collection (ATCC, Manassas, VA). Transfection was performed with Lipofectamine 2000 (11668027, Thermo Fisher Scientific, Waltham, MA) according to the manufacturer’s instructions. Experiments were performed 36 h after transfection.

Primary cultures of cortical and hippocampal neurons were prepared as previously described [34]. Briefly, individual cortical and hippocampal tissues were dissected from embryonic day E16.5 WT mice and placed in cold Hank’s balanced salt solution (HBSS) (Thermo Fisher Scientific). The tissues were digested with 0.025% trypsin for 25 min at 37 °C with gentle shaking every 5 min. After digestion, cells were dissociated using glass Pasteur pipettes gently pipetting up and down for 10 times. Cells were then resuspended in neurobasal medium (11668027, Thermo Fisher Scientific) supplemented with 2% B27 (17504044, Thermo Fisher Scientific) and 2 mM glutamax (35050061, Thermo Fisher Scientific). Cells were plated onto poly-*D*-lysine coated coverslips either at a low density of 200,000 cells/well (in a 12-well plate) for immunofluorescence or at a high density of 400,000 cells/well (in a 12-well plate) for immunoblotting analysis.

### Stereotaxic injection

Four-month-old WT and 5 × FAD mice were used for stereotaxic injection of AAV2/9. Briefly, mice were anesthetized with a gas mixture of oxygen (1 l/min) and 3% isoflurane for induction of anesthesia, fixed on a stereotaxic apparatus, and maintained under anesthesia throughout surgery at 1% isoflurane. AAV2/9 was injected unilaterally into the DG (AP − 1.3, ML ± 1.9, DV − 2.0) using a 1 μl needle (65460–02, Hamilton, Franklin, MA) at a rate of 50 nl/min with a total volume of 400 nl. The needle was kept in the injection site for 10 min after completion of each injection and then removed slowly. Virus was allowed to incubate for 4 weeks before any other procedures were performed.

### Immunoblotting

Immunoblotting was performed as described previously [35, 36]. Samples were homogenized and lysed with SDS sample buffer (63 mM Tris–HCl, 10% glycerol, and 2% SDS) containing a protease and phosphatase inhibitor cocktail (11836170001, Roche, Basel, Switzerland). Tissue lysates were sonicated followed by centrifugating at 14,000 × *g* for 10 min at 4 °C. Supernatant was collected. Protein concentrations were determined using the BCA Protein Assay (A65453, Thermo Fisher Scientific). Proteins (15–20 µg) were separated in an SDS-PAGE gel and transferred onto PVDF membranes (88518, Thermo Fisher Scientific). After blocking, the membranes were incubated overnight at 4 °C with corresponding primary antibodies (Table S1). The membranes were washed with PBST and incubated with secondary antibodies (Table S1) for one hour. Signals were detected with horseradish peroxidase–labeled antibodies and captured using the ChemiDoc Imaging System. Densitometric quantification of bands was analyzed using Image J (Version 1.54, NIH, Bethesda, MD).

### Immunofluorescent staining and confocal microscopy

Immunofluorescent staining was performed as previously described [34, 37, 38]. For *Drosophila*, thoraces of 3- to 5-day-old male flies were dissected and fixed in 4% paraformaldehyde in phosphate buffered saline (PBS). After thoraces were washed three times in PBS, muscle fibers were isolated and stained with rhodamine phalloidin (R415, Invitrogen™, Carlsbad, CA, 1:1000) in PBS containing 1% Triton X-100. For antibody staining, muscle fibers were permeabilized in PBS containing 0.1% Triton X-100, blocked with 5% normal goat serum in PBS, and incubated with primary and secondary antibodies diluted in 5% normal goat serum in PBS.

For mouse brain staining, 30-µm brain hippocampal slices were first washed three times with PBS and then permeabilized with 0.3% Triton X-100 in PBS at room temperature (RT). After blocking with 5% BSA in PBS for 60 min, free-floating slices were incubated with primary antibody at 4 °C overnight. The secondary antibodies were diluted in the blocking solution and incubated for 2 h at RT. Nuclei were counter-stained with DAPI. Slides were mounted with Fluoromount-G™ mounting medium (00–4958, Invitrogen™).

For immunostaining of cells or neurons, cells were fixed with 4% paraformaldehyde in PBS for 10 min, permeabilized in 0.1% Triton X-100 in PBS, and blocked in 5% BSA in PBS. Cells were incubated with primary antibody in a blocking solution (5% BSA in PBS) overnight at 4 °C, followed by incubation with secondary antibody for 1 h at RT. Nuclei were counterstained with DAPI. Slides were mounted in Fluoroshield mounting medium (Fluoromount-G™, Invitrogen™). All images were captured on a Zeiss LSM confocal microscope (Jena, Germany).

### Histological analysis

Mice were anesthetized with 4% isoflurane, and transcardially perfused with PBS followed by ice-cold 4% formaldehyde in PBS (pH 7.4). Brains were post-fixed in 4% formaldehyde in PBS at 4 °C overnight and then processed for frozen embedding following standard procedures. Coronal brain sections were cut at a thickness of 30 μm.

### Tunel staining

Briefly, fixed tissues (mouse brain slices and fly muscles) were permeabilized in 0.3% Triton X-100 in PBS for 1 h followed by 0.1% sodium citrate (0.3% Triton) for 30 min. Tunel staining was performed with an in situ cell death detection kit (12156792910, Roche, Basel, Switzerland) according to the manufacturer’s protocol. Nuclei were counter-stained with DAPI. Samples were mounted using Fluoromount-G™ mounting medium (00–4958, Invitrogen™) and imaged by a Zeiss LSM confocal microscope (Jena, Germany). Percentage (%) of cell death was calculated as the number of Tunel-positive cells divided by the DAPI-positive cells.

For quantification of different types of cell death in *Drosophila*, tif images were analyzed with a Fiji software (NIH, USA). Regions of interest were drawn and viewed with split channel function. Number of TUNEL-positive nuclei were counted and the intensity of mito-GFP was analyzed manually. At least 5 thoraces (*n* ≥ 5) and 3 brain slices of each mouse (*n* = 4) were analyzed for each genotype.

### Thioflavin S staining

Cryo-sectioned brain slices were incubated in filtered 1% Thioflavin-S in 50% ethanol for 8 min at RT. Slices were then washed with 50% ethanol, followed by two washes in PBS for 5 min each, mounted with coverslips in Fluoromount-G™ mounting medium (00–4958, Invitrogen™) and sealed with nail polish. Slides were stored at 4 °C until imaging.

### Real-time PCR

Total RNA isolated using Trizol reagent (15596026, Thermo Fisher Scientific) was converted to cDNA with the Verso cDNA Kit (K1691, Thermo Fisher Scientific) according to the manufacturer’s instruction. 2 × SYBR Green qPCR Master Mix (K0251, Thermo Fisher Scientific) was used for quantitative real-time PCR amplification with a CFX96 Real-Time PCR Detection System (Bio-Rad, Hercules, CA) and corresponding software (Applied Biosystems, Thermo Fisher Scientific). PCR was performed with 1 cycle at 50 °C for 2 min and 95 °C for 10 min, followed by 40 cycles of 95 °C for 15 s and 60 °C for 1 min. Gene expression was normalized to actin. Relative mRNA levels were calculated based on the comparative Ct method. Primers were as follows: C1qb (mouse) forward 5ʹ- GAGGTCTGGACACACCTGTTA-3ʹ, reverse 5ʹ- CTCCCCTTTAATCCCTGGAGT-3ʹ; C3 (mouse) forward 5ʹ- TGTCACTCACAGCCTTCGTC-3ʹ, reverse 5ʹ- ACTCCCCTGCCTTGTTGATG-3ʹ; IL-1β (mouse) forward 5ʹ- GCAACTGTTCCTGAACTCAACT-3ʹ, reverse 5ʹ- ATCTTTTGGGGTCCGTCAACT-3ʹ; IL-6 (mouse) forward 5ʹ- ATCCAGTTGCCTTCTTGGGACTGA-3ʹ, reverse 5ʹ- TAAGCCTCCGACTTGTGAAGTGGTʹ; TNF-α (mouse) forward 5ʹ- CATCTTCTCAAAATTCGAGTGACAA −3ʹ, reverse 5ʹ- TGGGAGTAGACAAGGTACAACCC −3ʹ; TGF-β1 (mouse) forward 5ʹ- CGCGTGCTAATGGTGGAC −3ʹ, reverse 5ʹ- ACTGCTTCCCGAATGTCTGAʹ; iNOS (mouse) forward 5ʹ- GTTCTCAGCCCAACAATACAAGA −3ʹ, reverse 5ʹ- GTGGACGGGTCGATGTCAC −3ʹ; β-actin (mouse) forward 5ʹ-GCCGGACTCATCGTACTCC −3ʹ, reverse 5ʹ-CCCCAGCATCAAAGGTG-3ʹ.

### ATP assay

ATP level was quantified using a commercial kit (ENLITEN® ATP Assay System, FF2000, Promega, Madison, WI). Total ATP levels were normalized to protein content.

### ROS production assay

Primary neurons were cultured in a poly-D-lysine-coated dish. Dihydroethidium (DHE) (Molecular Probes, Eugene, OR) was added to the dish at a final concentration of 20 μM in fresh medium and incubated for 50 min at 37 °C in a 5% CO_2_ humidified atmosphere. Following incubation, the dye solution was removed, and cells were gently washed twice with PBS. Nuclei were stained with Hoechst 33,342 for 30 min at RT. DHE fluorescence was measured by fluorescence microscopy with excitation/emission (Ex/Em) at 535 nm/610 nm. Images were acquired with consistent parameters for different samples. Fluorescence was quantified using the ImageJ software.

Flies were anesthetized and hemi-thoraces were dissected in cold *Drosophila* Schneider’s Medium (DSM). Hemi-thoraces were then incubated in a staining solution containing 5 μM MitoSOX Red in DSM for 12 min at RT. Then samples were washed twice with DSM (30 s each), quickly mounted in DSM and imaged within 10–15 min by a confocal microscope with same settings as those for primary neurons. Quantification of staining is done using Image J in which mean intensity for the MitoSOX stain was quantified.

### Tetramethylrhodamine ethyl ester (TMRE) mitochondrial membrane potential assay

Flies were anesthetized and hemi-thoraces were dissected in cold DSM. Hemi-thoraces were then incubated in 100 nM TMRE in DSM, for 15 min at RT. Samples were rinsed twice (30 s each) with 25 nM TMRE in DSM. Samples were quickly mounted in 25 nM TMRE in DSM onto the slide and imaged within 15–20 min by confocal microscope with same settings as those for ROS production assay. Quantification of TMRE staining was conducted using Image J.

### Analysis of *Drosophila* abnormal wing phenotypes

For analysis of abnormal wing phenotype, over 50 flies were placed per vial. Flies with both wings held-up or drooped were counted.

### Mouse behavioral analysis

Behavioral tests were conducted in the following sequence: Open field test, Y maze test, novel-object recognition test, elevated plus maze test, and Morris water maze. All tests were conducted between 9:00 a.m. and 6:00 p.m. Male mice of 5 months old were bred and housed in the testing room for a minimum of 2 weeks prior to the initial test to allow acclimatization to the environment. Each test was separated by a rest period of at least 24 h. The experimenter was blinded to group assignments during all tests.

Open field test was employed to evaluate the general activity level and anxiety of mice. Briefly, a mouse was gently placed into the open-field apparatus (measuring 72 cm × 72 cm × 40 cm) and allowed to freely explore the area for 10 min. The total distance traveled, speed, time spent in the open field, distance traveled in the central area (36 cm × 36 cm), and number of entries into the central area were analyzed.

The Y-maze was used to assess cognitive ability and spatial working memory. The apparatus comprised three radial arms positioned at equal angles (120°). At the beginning of each trial, a mouse was placed in the central area and permitted to freely navigate the maze for 8 min. Efficient behavior was characterized by consecutive entries into all three arms, with each arm being visited in succession without repeating the same arm until all three had been explored. The number of sequential entries into three distinct arms of the maze was counted.

The novel-object recognition test assesses cognition, specifically spatial memory and discrimination. The test was conducted in an open field arena, where animals were first habituated for 10 min, during which they were allowed to freely explore the empty arena. On the second day, animals were placed at the center of the arena in the presence of two objects. Objects were secured to the floor with tape. On the third day, one of the objects was replaced with a novel object featuring different characteristics, such as shape, color, size, or texture. The objects were positioned approximately equidistant from the center of the arena. Exploratory behavior, including sniffing and touching, was considered an indication of recognition. The arena and objects were thoroughly cleaned between trials using a 30% alcohol (*v*/*v*) solution to minimize olfactory cues. Mouse behavior was recorded using a camera mounted above the arena and scored for the exploration time of the new object and the frequency of interactions with each of the objects.

The elevated-plus maze test evaluates anxiety, which was a 6-min trial in a dimly lit room. The maze was elevated 60 cm above the floor and composed of two open arms and two closed arms, each measuring 5 × 5 cm^2^. A mouse was placed in the center of the maze facing one open arm and allowed to freely explore the maze. The time spent and the distance traveled in the different arms were recorded with AniLab software (Ningbo, China).

The Morris water maze test evaluates spatial learning and memory. The test apparatus consisted of a circular pool (120 cm in diameter and 50 cm in height) filled with opaque water, and a white plastic platform submerged 1 cm below the water surface in one of the quadrants. The pool was divided into four quadrants, and the location of the hidden platform remained fixed throughout the training process. Prior to training, mice underwent a pretraining day to familiarize them with the environment without platform for 60 s. During the subsequent five training days, mice were allowed to swim freely in the pool to locate the platform. Mice that failed to locate the platform within 60 s were manually guided to the platform. The mice were allowed to stay on the platform for 10 s. Three trials were performed on each training day, with a 30 min interval between trials. The probe tests were conducted one hour and 24 h after the final training session, respectively, during which the platform was removed and mice were allowed to swim for 60 s. The latency, the swimming paths, number of crossings, and percentage of time spent in each quadrant were recorded using a video camera system.

### Mitochondrial morphology analysis

For *Drosophila* muscle, mitochondria were labelled by mito-GFP. Over 10 flies/group and 3–5 pictures of different microscopic fields from each fly were analyzed. The average mitochondrial size (μm^2^) was calculated to determine the morphological changes.

### Transmission electron microscopy (TEM)

Fly thoraces dissected from 3-day-old male flies or mouse hippocampal tissues were fixed in paraformaldehyde/glutaraldehyde, then post-fixed in osmium tetraoxide, dehydrated in ethanol, and embedded in Epon. After polymerization of Epon, blocks were sectioned to generate 70-nm-thick sections using a diamond knife on a microtome (Leica, Wetzlar, Germany). The sections were stained with uranyl acetate and lead citrate. Digital images were obtained on a Tecnai G2 Spirit by FEI equipped with an Eagle 4 k HS digital camera. Aspect ratio (AR) (length-to-width ratio) of mitochondria was calculated as follows: AR = major axis/minor axis.

### Respiration measurements using Seahorse XFe96 analyzer

Primary cortical and hippocampal neurons were plated onto pre-coated Matrigel XF 96-well plates (Agilent, Santa Clara, CA) at a density of 40,000 cells/well in 100 μl growth medium, and cultured at 37 °C in a 5% CO_2_ atmosphere for 10 days. On the day of experiment, cells were washed twice and pre-incubated for 45 min in Seahorse DMEM basal medium (pH 7.4) (Agilent) supplemented with 10 mM glucose, 1 mM pyruvate, and 2 mM glutamine, allowing medium temperature and pH to reach equilibrium before the initial rate measurement. Cells were ensured to be incubated in a CO_2_-free environment. Mitochondrial activities were assessed at baseline and again following addition of the electron transport chain accelerator ionophore 4-(trifluoromethoxy) phenylhydrazone (FCCP, 4 μM), which induces maximal oxygen consumption rate (OCR). Finally, respiration was halted by adding the electron transport chain inhibitors 0.5 μM rotenone and 0.5 μM Antimycin A. The omission of Oligomycin, which impacts electron flow through the ETC, was implemented in experiments to account for potential influences on the estimation of maximal uncoupled respiration. Data were normalized to cellular protein levels, and basal OCR and maximal OCR were evaluated.

### RNA extraction, sequencing and analysis

Total RNA was extracted from tissues utilizing a Trizol-based method (15596026 Thermo Fisher Scientific). RNA concentration and integrity were assessed using a NanoDrop spectrophotometer (Thermo Fisher Scientific) and an Agilent 2100 Bioanalyzer (Agilent, Santa Clara, CA), respectively. Poly(A) RNAs were isolated from total RNA using oligo-dT magnetic beads (S1419S, New England Biolabs, Ipswich, MA). Poly(A) RNAs were subsequently fragmented into approximately 200 bp fragments. Random hexamers were employed for cDNA synthesis followed by converting into double-stranded cDNA. The resulting cDNA library was sequenced on an Illumina HiSeq platform (San Diego, CA), generating 150 bp paired-end reads. Raw sequencing data underwent quality control analysis using FastQC and were aligned to a reference genome (UCSC mouse genome version mm10) using alexdobin/STAR (Version 2.4, Spliced Transcripts Alignment to a Reference © Alexander Dobin, 2009–2024). Gene expression levels were quantified via featureCounts-2.0.3 (featureCounts is available under GNU General Public License as part of the Subread (http://subread.sourceforge.net) or Rsubread (http://www.bioconductor.org) software packages). Differential gene expression analysis was conducted using DESeq2 (Version 3.19, Bioconductor).

### Co-immunoprecipitation and liquid chromatography-mass spectrometry/ mass spectrometry (LC–MS/MS) analysis

Thoracic tissues were dissected from over 150 male flies of 3 to 5 days age. After homogenization, tissues were lysed on ice for 1 h in Co-IP lysis buffer (50 mM Tris–HCl, pH 7.5, 150 mM NaCl, 1 mM EDTA, 1% Triton X-100, supplemented with protease and phosphatase inhibitors). For cells, HEK293 cells were transfected with indicated plasmids, harvested 36 h after transfection and lysed with the Co-IP buffer on ice for 1 h. After centrifugation at 13,000 rpm for 20 min at 4 °C, supernatants were collected and immunoprecipitated by M2 (anti-Flag antibody)-Trap agarose beads (A2220, MilliporeSigma, Darmstadt, Germany) at 4 °C overnight. The bound proteins were washed, eluted with SDS lysis sample buffer and analyzed by LC–MS/MS.

LC–MS/MS was performed essentially as previously described [39]. Briefly, samples were digested with trypsin at 37 °C overnight, desalted by reversed-phase C18 Sep-Pak cartridge (Millipore, Temecula, CA). Samples were loaded on an EASY-nLC1000 LC system (Thermo Scientific) coupled to the Q-Exactive mass spectrometer (Thermo Scientific). The peptides were dissolved in 0.1% formic acid (FA) with 2% acetonitrile (ACN) and separated on a RP-HPLC analytical column (75 μm × 15 cm) packed with 3 μm C18 beads using a gradient ranging from 12% to 32% buffer B (98% acetonitrile and 0.1% acetic acid) for 120 min. The resolution for MS was set to 70,000 and that for MS/MS was set to 17,500. The raw files were processed by MaxQuant software (version 1.4.1.2) with searches against the Uniprot human database (20,311 entries, downloaded August 2020) and Uniprot *Drosophila* database (3596 entries, downloaded September 2020).

### Functional enrichment analysis

Significant differential expression genes (DEGs) and co-immunoprecipitated proteins were analyzed using the Metascape online meta-analysis tools for functional clustering. For DEGs and co-immunoprecipitated proteins identified in HEK293 cells, enriched annotations from Gene Ontology Biological Processes, Reactome gene sets, Wiki Pathways, Canonical Pathways, KEGG Pathways and PANTHER Pathways were included. Clustering parameters were set as recommended: minimal overlap 3, minimal enrichment ratio 1.5, *P*-value < 0.01. Co-immunoprecipitated proteins identified in *Drosophila* tissues were analyzed based on *Drosophila*-derived functional annotations using the same parameters. Top 20 enrichment clusters were shown as column plot using the ggplot2 package. For co-immunoprecipitated proteins, cellular distributions were also analyzed by Metascape online tools using the same clustering parameters. All significant clusters for *Drosophila* and top 10 clusters for HEK293 are shown as point plot by the ggplot2 package.

### Protein–protein interaction (PPI) network construction

The PPI network of co-immunoprecipitated proteins was constructed using the Search Tool for the Retrieval of Interacting Genes (STRING, version 11.0, https://string-db.org/) online database. Interaction scores > 0.9 (highest confidence) were regarded as significant interaction. Full STRING network containing interactions derived from experimental evidence by this cut off is shown. The interaction network was constructed by the Cytoscape software.

### Statistical analysis

Data analysis was performed using Prism 9 software (GraphPad, La Jolla, CA). Two-tailed Student’s *t* test was used to determine the significance of difference between two groups. Statistical significance between multiple groups was analyzed with one-way ANOVA followed by Tukey’s test. The difference between treatment groups and their controls was assessed using a Multiple-way ANOVA with Dunnett’s tests. All error bars indicate SEM. Quantitation was performed in a double-blinded manner.

## Results

### Expression of SARS-CoV-2 M causes apoptotic cell death in *Drosophila* and primary mouse neuronal cultures

**Fig. 1:**
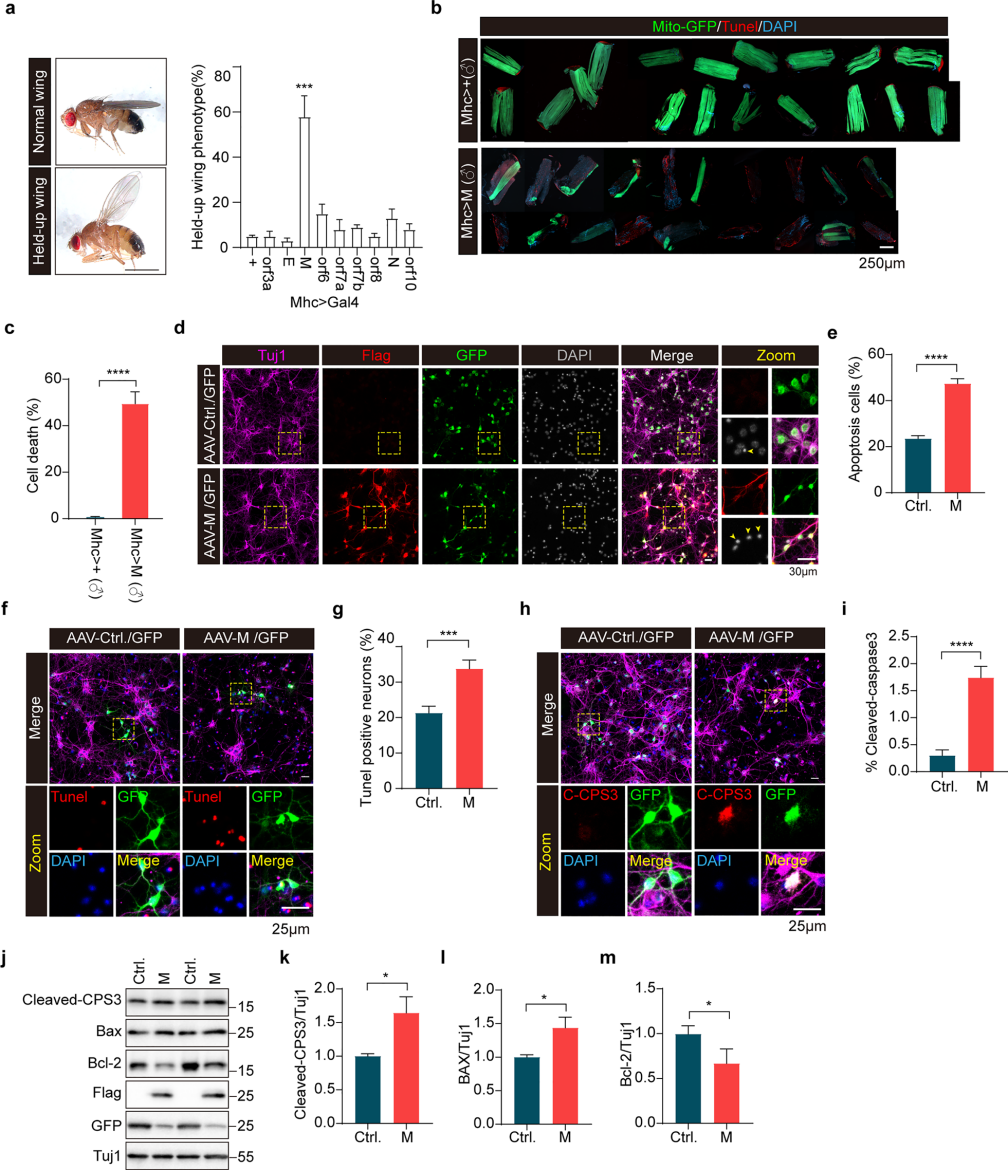
Expression of SARS-CoV-2 M results in apoptotic neuronal death in vitro. **a** Abnormal wing phenotype of *Drosophila* expressing SARS-CoV-2 proteins. Left, a representative microscopic view (left panels) of an adult male fly showing a “held-up” wing phenotype (lower) compared to a normal fly (upper). Right, quantitative analysis of the “held-up” wing phenotype in flies expressing SARS-CoV-2 proteins.* n* > 200 flies/group. ***P < 0.001. Mean ± SEM, one-way ANOVA followed by Tukey’s test. **b, c** Indirect flight muscle (IFM) of flies expressing M underwent apoptotic cell death. Confocal microscopic sections of IFM preparations from 3- to 5-day-old male (♂) flies expressing either Mhc-gal4 (Mhc > +) or Mhc-gal4-driven SARS-CoV-2 M (Mhc > M) stained for Tunel dye. Each piece of IFM was dissected from one fly. Cell death was quantified for each genotype.* n* > 20 flies/group. ****P < 0.0001. Data from three independent experiments are expressed as mean ± SEM. Unpaired Student’s *t-*test **d, e** Expression of SARS-CoV-2 M leads to apoptotic cell death in primary neuronal cultures. The cultured primary cortical neurons were infected with AAV virus expressing either M or control GFP (Ctrl) for 4 days. Enlarged images are shown in the rightmost column. Flag staining shows M expression; GFP staining shows virus infection. The percentage of apoptotic neurons with nuclear shrinkage was analyzed, *n* > 100 neurons/group. ****P < 0.0001. Results from three independent experiments, mean ± SEM, unpaired Student’s *t-*test. **f–i** Apoptotic neuronal death induced by M expression. Tunel staining (**f)** and immunofluorescent staining of cleaved caspase-3 (C-CPS3) (**h)** were performed for primary cortical neurons infected with AAV virus expressing either M or control GFP (Ctrl) for 4 days. Percentages of Tunel^+^ neurons and C-CPS3^+^ neurons were analyzed. **g**, **i**,* n* > 100 neurons/group. ***P < 0.001, ****P < 0.0001. Results from three independent experiments, mean ± SEM, unpaired Student’s *t-*test. **j–m** Immunoblotting for apoptosis-related proteins in primary cortical neurons infected with AAV virus expressing either M or control GFP (Ctrl) for 4 days. Tuj1 was used as a loading control. The relative expression levels of active-caspase-3 (**k)**, Bax (**l)**, and Bcl-2 (**m)** were quantified. *P < 0.05. Results from three independent experiments, mean ± SEM, unpaired Student’s *t-*test

To explore cellular mechanisms of SARS-CoV-2 pathogenesis, we expressed flag-tagged SARS-CoV-2 proteins, including three structural proteins (E, M, and N) and six accessory proteins (ORF3a, ORF6, ORF7a, ORF7b, ORF8, and ORF10), in flight muscles of *Drosophila* using a mhc-gal4 driver (Fig. S1a). Expression of viral proteins in S2 cells and *Drosophila* thoracic muscle tissues was verified by immunoblotting using an anti-flag antibody. All 9 proteins were detected in S2 cell lysates with predicted molecular weight (Fig. S1b). However, only 4 proteins (ORF3a, M, ORF7b, and N) were detected in the muscle tissue lysates (Fig. S1c). Immunostaining using an anti-flag antibody detected ORF3a, E, M, N, and ORF10 in flight muscle tissues (Fig. S1d). Nevertheless, mRNA expression of all 9 proteins was detected at a similar level using quantitative RT-PCR (Fig. S1e). Ubiquitous expression of 9 proteins driven by actin-gal4 showed that expressing M or ORF6 resulted in embryonic lethality of *Drosophila*. Expression of ORF3a driven by actin-gal4 also resulted in embryonic lethality in male flies (Data not shown). Flies expressing M in indirect flight muscles (IFM) driven by mhc-gal4 exhibited a held-up posture of wings (Fig. 1a). The abnormal wing phenotypes (including a held-up posture and crumpled wings) were confirmed in flies expressing M under the mef2-Gal4 driver (another driver for muscle tissue expression) (Fig. S2a–c). We next dissected IFM from flies expressing M for further characterization. Muscles expressing M showed increased cell death by Tunel staining and loss of mitochondria (Fig. 1b, c). Surprisingly, cell death induced by M expression was significantly higher in male flies than in female flies (Fig. S2d, e). Consistently, rate of abnormal wing phenotypes detected in flies expressing M driven by mef2-Gal4 was significantly higher in male flies than in female flies (Fig. S2a–c). We also noticed that the amount of cell death was correlated with the severity of mitochondrial damage (Fig. 1b, c, Fig. S2d, e). Thus, M induces sex-dependent abnormal phenotypes in *Drosophila*.

To characterize M-induced cell death, we individually overexpressed two anti-apoptotic factors, the viral caspase inhibitor p35 or the *Drosophila* inhibitor of apoptosis 1 (DIAP1), in muscle tissues of flies expressing M. Under control of mhc-gal4, expression of p35 or DIAP1 alone did not induce cell death in fly muscle tissues. However, when M was expressed, p35 and DIAP1 expression both significantly suppressed muscle cell death induced by M (Fig. S3a, b). This suggested that M induces apoptotic cell death in *Drosophila*.

Many studies have shown the infiltration of the SARS-CoV-2 virus into the brains of infected individuals [40, 41]. We next infected mouse cortical neuronal cultures with AAV virus for M expression (Fig. 1d). Expression of M resulted in a significant reduction in the number of neurons (Fig. 1d, e). Tunel staining and immunostaining for cleaved-caspase3 showed increased signals in neuronal cultures expressing M compared with control infections (Fig. 1f–i). Moreover, M expression resulted in Bcl-2 downregulation, Bax upregulation, and caspase-3 activation in primary cortical cultures (Fig. 1j–m). We also used SARS-Cov-2 N protein as a control in primary neuron models. The results showed that N expression did not induce neuronal death in our experiments (Fig. S4a–d).

Minks can be infected by SARS-CoV-2 and transmit the virus to humans [42]. To explore whether SARS-CoV-2 infected mammals show the mitochondrial phenotypes detected in flies expressing M, 13-month-old female minks were intranasally inoculated with 107 PFU of strain SARS-CoV-2/HRB25/human/2020/CHN (GISAID access no. EPI_ISL_467430). Minks mock inoculated with PBS were included as negative controls. Four days after inoculation, the animals were euthanized and sacrificed. Lung and nasal turbinate tissues were collected for TEM analysis. Viral infection was verified by TEM (Fig. S5). Disrupted mitochondrial cristae and loss of cristae were observed in both lung tissues and nasal turbinate tissues of infected animals, but not in control animals (Fig. S5a, b). Apoptotic bodies and macrophage-engulfed apoptotic cells were also seen in lung tissues (Fig. S5c) and nasal turbinate tissues of infected animals but not in control animals. These results suggest that SARS-CoV-2 infection causes mitochondrial abnormality and apoptotic cell death in mammals.

### Expression of SARS-CoV-2 M impairs mitochondrial function in *Drosophila* and primary neurons

**Fig. 2:**
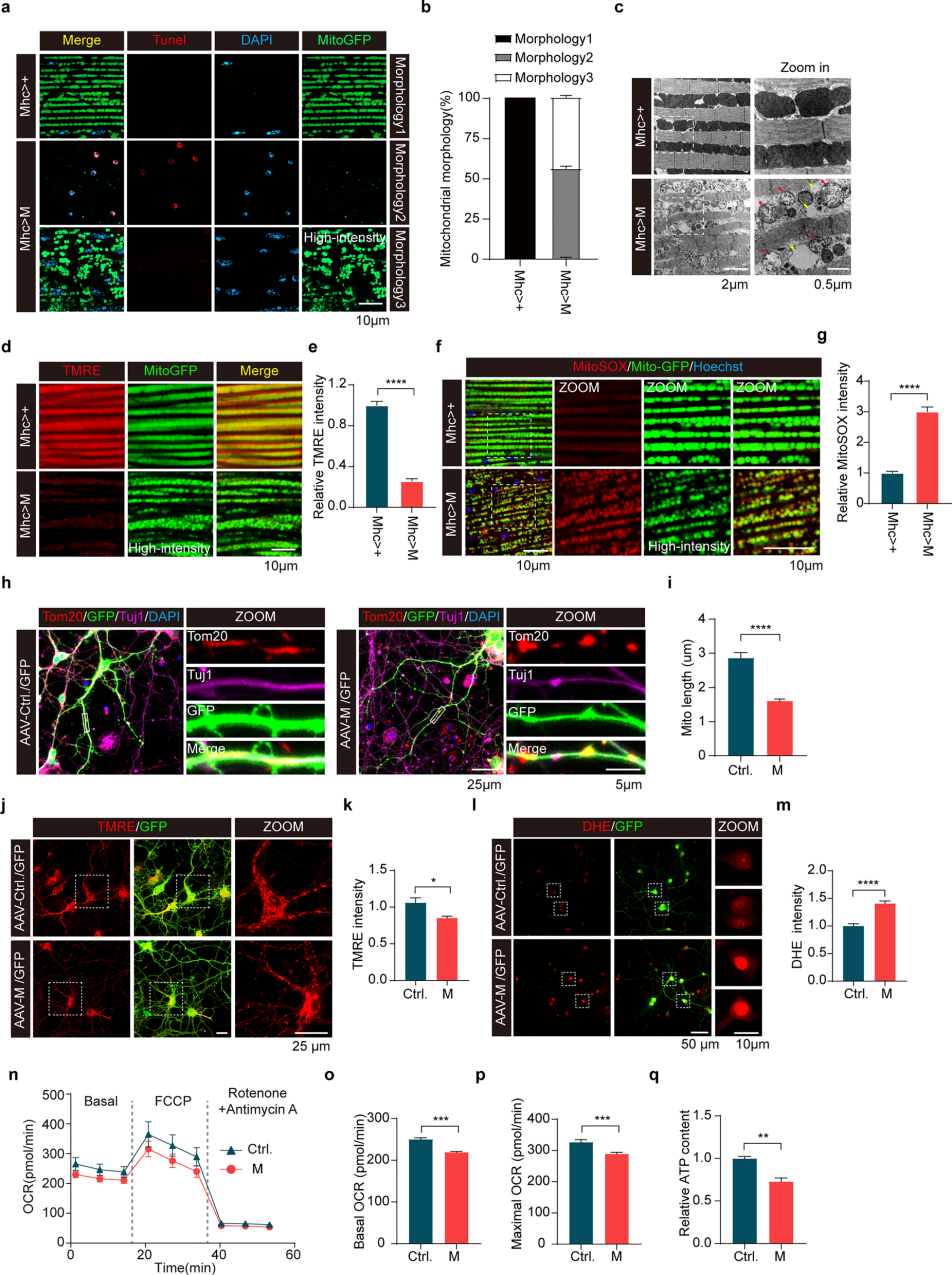
Expression of SARS-CoV-2 M impairs mitochondrial function in *Drosophila* and primary neurons.** a**, **b** Confocal microscopic sections of indirect flight muscle (IFM) preparations from 3- to 5-day-old male flies expressing either Mhc-gal4 (Mhc > +) or Mhc-gal4-driven SARS-CoV-2 M (Mhc > M) followed by Tunel staining. Three different types of mitochondrial phenotype in each genotype were quantified.* n* > 10 flies/group. Mean ± SEM. **c** TEM analysis of IFM tissues from 3- to 5-day-old male flies expressing Mhc-gal4 (Mhc > +) or Mhc-gal4-driven SARS-CoV-2 M (Mhc > M). Red arrowheads: damaged cristae in mitochondria. Yellow arrows: loss of cristae in mitochondria. **d, e** Confocal microscopic sections of IFM preparations from 3- to 5-day-old male flies expressing either Mhc-gal4 (Mhc > +) or Mhc-gal4-driven SARS-CoV-2 M (Mhc > M) stained for TMRE and quantitation of TMRE intensity. TMRE staining detects mitochondrial membrane potential. ****P < 0.0001. Results were from three independent experiments, mean ± SEM, unpaired Student’s *t-*test **f**, **g** MitoSOX staining of fly IFM tissues from 3- to 5-day-old male flies expressing either Mhc-gal4 (Mhc > +) or Mhc-gal4-driven SARS-CoV-2 M (Mhc > M) and quantitation of MitoSOX intensity. ****P < 0.0001. Results were from three independent experiments, mean ± SEM, unpaired Student’s *t-*test. **h**, **i** Mitochondrial fragmentation in dendrites of neurons expressing M. Primary cortical neurons were infected with AAV virus expressing either M (right panels) or control GFP (Ctrl, left panels) for 4 days followed by mitochondrial morphological analysis. Mitochondrial length in neuronal dendrites was measured and quantified. Three to 5 secondary dendrites per neuron, > 50 neurons per indicated group were analyzed. ****P < 0.0001. Results were from three independent experiments, mean ± SEM, unpaired Student’s *t-*test. **j**, **k** TMRE staining of primary cortical neurons infected with AAV virus expressing either M or control GFP (Ctrl) for 4 days. TMRE intensity in each neuron was analyzed. *P < 0.05. Results were from three independent experiments, mean ± SEM, unpaired Student’s *t*-test. **l**, **m** DHE staining to detect ROS generation. DHE intensity in each neuron was analyzed.* n* > 100 cells per indicated group were analyzed. ****P < 0.0001. Results were from three independent experiments, mean ± SEM, unpaired Student’s *t*-test. **n–p** Real-time oxygen consumption rate (OCR) was measured in 10 DIV primary neurons infected with AAV virus expressing either M or control GFP (Ctrl) for 4 days using a Seahorse XF analyzer. Sensor cartridge was incubated for 60 min prior to experiment in XF assay medium and then was consecutively injected with CCCP followed by antimycin A/rotenone. Continuous OCR values (pmoles/min) are shown (**n)**. The basal (**o)** and maximal (**p)** OCR are presented. ***P < 0.001, ****P < 0.0001. Results were from three independent experiments, mean ± SEM, unpaired Student’s *t-*test. **q** ATP contents of neurons infected with AAV virus expressing either M or control GFP (Ctrl) for 4 days were determined and normalized against the protein contents. **P < 0.01. Results were from three independent experiments, mean ± SEM, unpaired Student’s* t*-test

To determine the effects of SARS-CoV-2 proteins on mitochondria, we co-expressed mito-GFP and SARS-CoV-2 proteins in the IFM of *Drosophila*. Remarkable decreases of mito-GFP fluorescence intensity were observed in muscles expressing M compared to control muscles expressing mhc-gal4 alone. Notably, muscles expressing M showed mitochondrial fragmentation compared to muscles expressing the other 8 SARS-CoV-2 proteins (Fig. S6a). Two different types of phenotypes were observed in fly muscles expressing M, including few mitochondria with marked cell death (Morphology 2), and fragmented mitochondria with no cell death (Morphology 3). Male flies expressing M showed Morphology 2 and 3, while Morphology 1 (normal mitochondria with no cell death) was hardly detected (Fig. 2a, b). ATP content assay demonstrated significantly reduced ATP content in muscles expressing either M or ORF6 (Fig. S6b). TEM analysis verified mitochondrial fission with low electronic density and abnormal cristae in muscles expressing M (Fig. 2c). In contrast, no abnormal mitochondrial morphology was detected in muscles expressing the other 8 SARS-CoV-2 proteins (Fig. S6c). There was a correlation between mito-GFP fluorescence intensity and Tunel staining signaling in IFM of flies expressing M. The weaker the mito-GFP fluorescence signal, the more apoptotic cells it contains in muscle with M expression.

The mitochondrial membrane potential (ΔΨm) generated by proton pumps is an essential component in the process of energy storage during oxidative phosphorylation. TMRE staining revealed that expression of M markedly decreased the mitochondrial potential in fly IFMs compared to that of control IFMs expressing mhc-gal4 alone (Fig. 2d, e). Consistently, M expression markedly induced ROS production and accumulation in mitochondria in IFMs compared to controls expressing mhc-gal4 alone (Fig. 2f, g). These results suggest that M protein causes impairment of mitochondrial functions. Consistently, M expression induced a notable increase of mitochondrial fragmentation, a decrease of mitochondrial membrane potential, and an increase in ROS accumulation in primary cultured neurons compared to control neurons (Fig. 2h–m). Moreover, the mitochondrial oxygen consumption and cellular ATP levels in primary neurons were markedly decreased in neurons expressing M compared to that of control neurons (Fig. 2n–q). Together, results suggest that M expression causes structural abnormalities and functional impairment in mitochondria in muscles of flies and mouse neurons.

### SARS-CoV-2 M induces hippocampal atrophy and apoptotic neuronal death in mouse brains

**Fig. 3:**
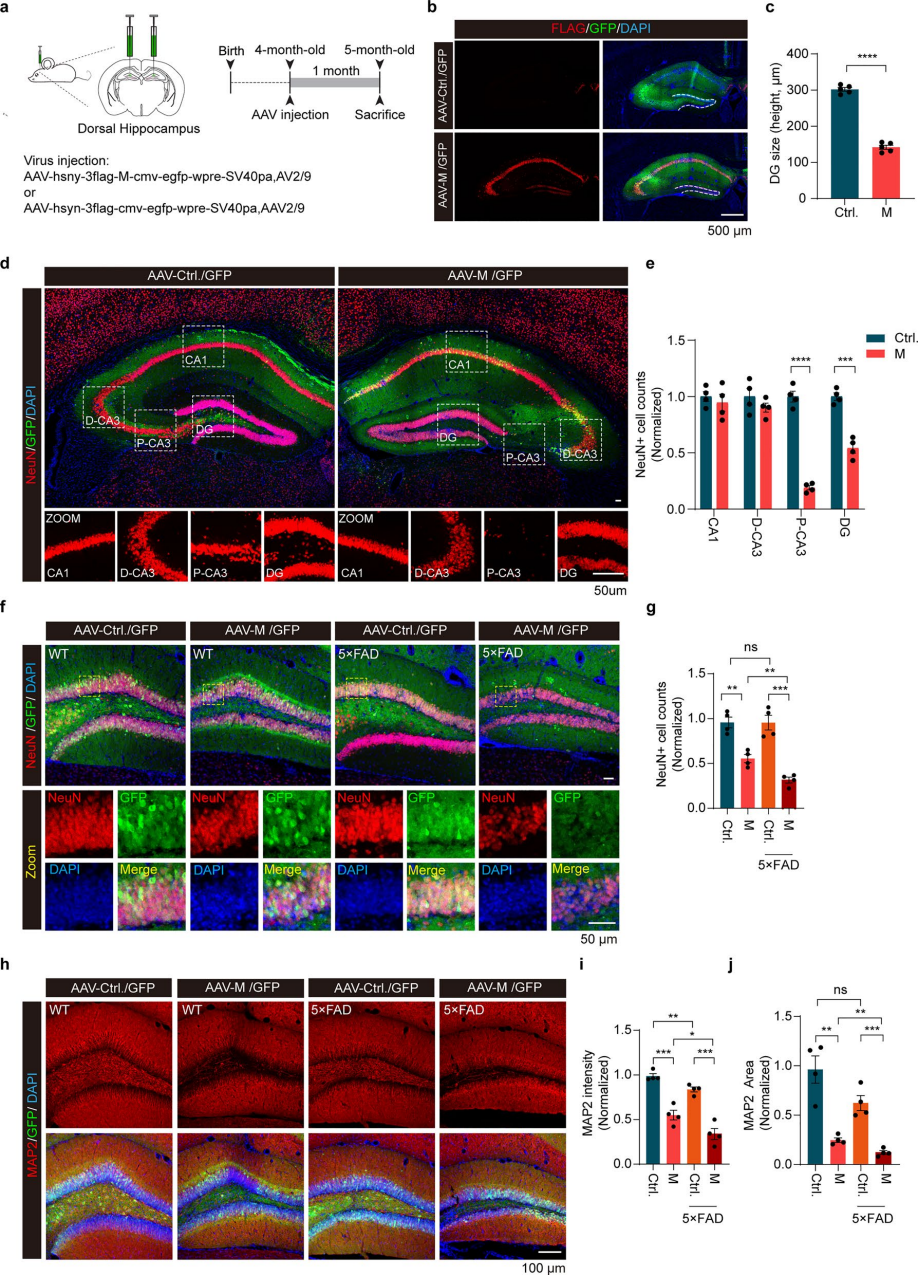
M induces hippocampal atrophy in WT and 5×FAD mice. **a** Schematic illustration of AAV-mediated ectopic expression of M in mouse dorsal hippocampus. **b**, **c** Immuno-staining of hippocampal sections of mice expressing either M or control GFP. Red: Flag to detect M; Green: GFP to show AAV infection; Blue: DAPI to stain nuclei. The height of dorsal DG in the hippocampus was quantified.* n* > 4 mice per indicated group. ****P < 0.0001*.* Results were from three independent experiments, mean ± SEM, unpaired Student’s *t*-test. **d**, **e** Confocal images of NeuN-positive neurons in the hippocampus expressing either M or control GFP. The numbers of neurons in CA1, distal CA3 (D-CA3), proximal CA3 (P-CA3), and dorsal DG were quantified. ***P < 0.001, ****P < 0.0001. Results were from three independent experiments, mean ± SEM, unpaired Student’s *t-*test. **f**, **g** Confocal images of NeuN-positive neurons in the hippocampus of wild-type (WT) and AD mice expressing either M or control GFP (Ctrl). The numbers of neurons in dorsal DG were quantified. **P < 0.01, ***P < 0.001*,* ns, no significance. Results were from three independent experiments, mean ± SEM, multi-way ANOVA followed by Dunnett’s test. **h–j** Confocal images of MAP2-positive neurons in the hippocampus of WT and AD mice expressing either M or control GFP (Ctrl) (**h)**. The immunoreactivity (**i)** and the area (**j)** of MAP2 staining were quantified. *P < 0.05, **P < 0.01, ***P < 0.001, ns, no significance. Results were from three independent experiments, mean ± SEM, multi-way ANOVA followed by Dunnett’s test

COVID-19 patients present with cognitive impairments like memory deficits, attention problems, coordination issues, and mood disturbances [43]. The hippocampus plays an essential role in the formation and consolidation of new memories that rely on the integrity of synaptic circuitry. We next injected AAV vector encoding M under the neuron-specific expression promoter (AAV-hsyn-3xflag-M-cmv-EGFP) into the left and right hippocampi of 4-month-old mice to induce expression of M in the CA1-3 and dentate gyrus (DG) regions (Fig. 3a). AAV-Control-GFP was injected as a control. Expression of the injected viruses was confirmed by immunostaining with an anti-flag antibody (to detect flag-tagged M) and an anti-GFP antibody (to detect EGFP) (Fig. 3b). Results showed significant atrophy in the DG region of mice expressing M compared to that expressing GFP alone at 1 month after injection (Fig. 3b, c). Specifically, M induced neurodegeneration in the hippocampal formation, particularly in the DG and proximal CA3 (P-CA3) regions. In contrast, the CA1, CA2, and distal CA3 (D-CA3) regions remained unaffected (Fig. 3d, e). Remarkably, cells between DG molecular layers were very vulnerable to M-induced degeneration (Fig. 3d, e). Immunostaining of neuronal markers NeuN and MAP2 revealed a significant decrease of NeuN-positive and MAP2-positive cells in the DG region expressing M compared to that expressing either N or control GFP alone (Fig. 3f–j, Fig. S7a–c). Consistently, M expression for 4 months further increases neurodegeneration in DG region, suggesting that M induced progressive neuronal loss in the mouse brain (Fig. S7d, e).

**Fig. 4:**
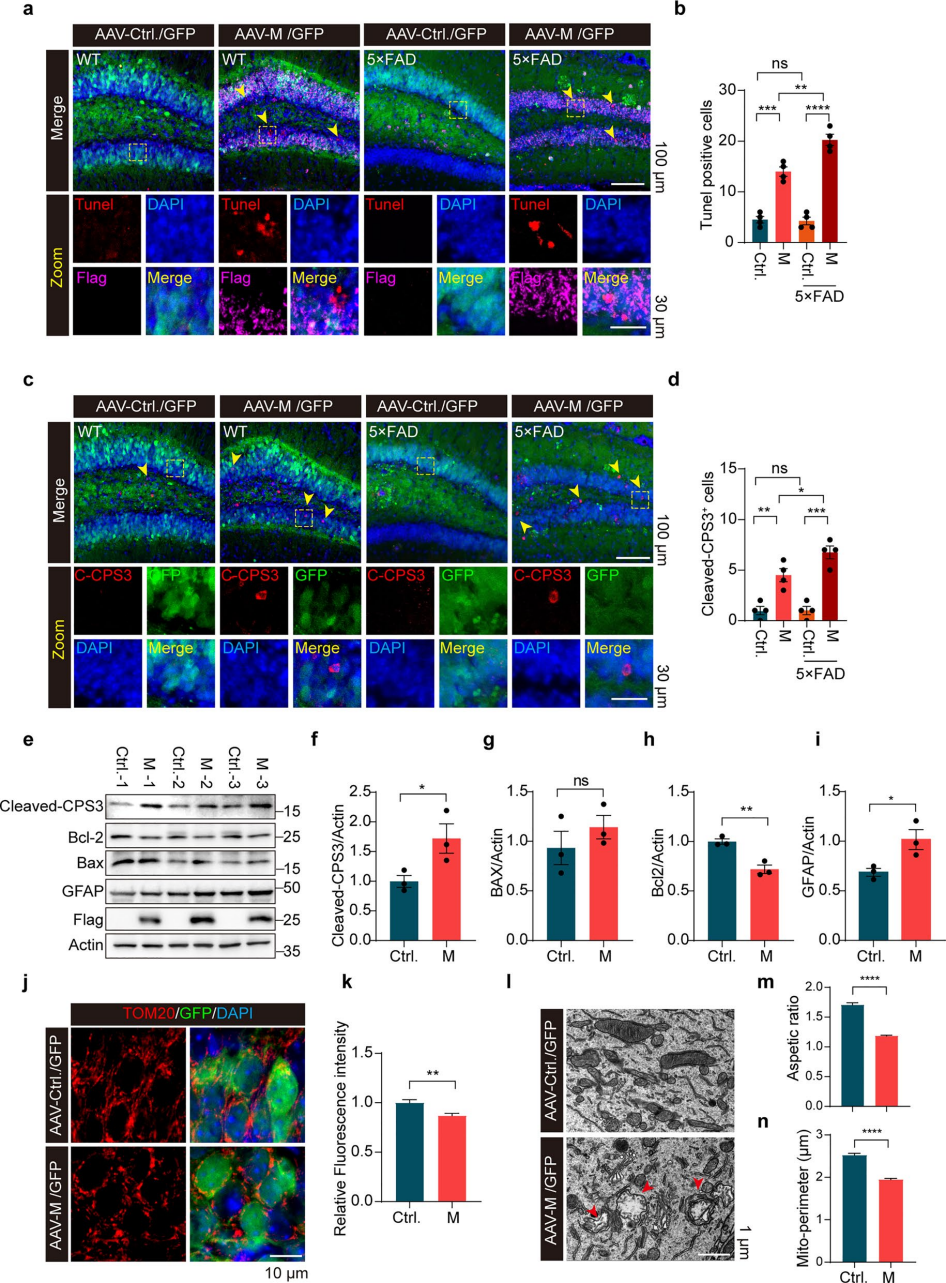
SARS-CoV-2 M induces apoptotic neuronal death in WT and 5×FAD mice. **a**, **b** Immunofluorescence images of the dentate gyrus. The numbers of Tunel-positive neurons were quantified.* n* = 4 mice/group. **P < 0.01, ***P < 0.001, ****P < 0.0001. Results from three independent experiments are expressed as mean ± SEM. Multi-way ANOVA followed by Dunnett’s test. **c, d** Caspase-3 staining of mouse hippocampus expressing M. The numbers of cleaved-caspase 3 (CPS3)^+^ neurons in the indicated groups were analyzed.* n* = 4 mice/group. *P < 0.05, **P < 0.01, ***P < 0.001. Results from three independent experiments are expressed as mean ± SEM. Multi-way ANOVA followed by Dunnett’s test. **e–i** Immunoblotting analysis of apoptosis-related proteins. Samples from three control mouse hippocampi (Ctrl.) and three mouse hippocampi expressing M (**e)**. The relative expression of cleaved-CPS3 (**f)**, Bcl-2 (**h)**, Bax (**g)**, and GFAP (**i)** was quantified. *P < 0.05, **P < 0.01. Results were from three independent experiments, mean ± SEM, unpaired Student’s *t-*test. **j**, **k** Mitochondria staining in mouse hippocampus expressing M and quantification of the relative fluorescence intensity of Tom20 in neurons. Three to five fields per mouse. > 50 cells in each indicated group were analyzed. **P < 0.01. Mean ± SEM, unpaired Student’s *t-*test. **l–n** TEM analysis of mouse hippocampus expressing M. Aspect ratio (**m)** and mitochondria perimeter (**n)** were quantified.* n* = 3 mice/group. ****P < 0.0001. Mean ± SEM, unpaired Student’s *t-*test. ns, no significance

A previous study reported that COVID-19 survival is associated with an increase in risk of longitudinal cognitive decline [44]. Therefore, we also included an AD mouse model (5 × FAD) in this study [45]. Results showed that M expression further reduced NeuN-positive cells and decreased MAP2-positive area in 5-month-old 5 × FAD mice (Fig. 3f–j). Tunel assay and immunostaining for cleaved caspase-3 showed increased detection of Tunel-positive and cleaved caspase-3-positive cells in the DG of mouse hippocampi expressing M compared to that expressing control GFP (Fig. 4a–d). Consistently, immunoblotting analysis of lysates derived from WT mouse hippocampi showed increased levels of cleaved caspase-3, Bax, and GFAP and decreased Bcl-2 with M expression (Fig. 4e–i). Remarkably, the 5-month-old 5 × FAD mice expressing M had significantly more Tunel-positive cells and cleaved caspase-3-positive cells in the DG region than the age-matched WT mice expressing M (Fig. 4a–d). In contrast, both WT mice and 5 × FAD mice expressing control GFP exhibited few Tunel-positive and cleaved caspase-3-positive cells in the DG region (Fig. 4a–d). Furthermore, Tom20 staining was significantly reduced in the DG region of WT mice expressing M compared to mice expressing GFP alone (Fig. 4j, k). TEM analysis showed increased mitochondrial fission in the DG of mice expressing M (Fig. 4l–n). Damage to mitochondrial cristae was also observed (Fig. 4l). These results suggest that M promotes mitochondrial fragmentation and induces apoptotic neuronal death in DG. Intriguingly, neurons in DG of 5 × FAD mice are more sensitive to M than neurons in WT mice.

### Expression of M promotes formation of Aβ dense plaques in 5 × FAD mouse brains

Since the DG region of 5 × FAD mice is more sensitive to M-induced neuronal death, we next analyzed Aβ deposition in these mice. AAV-M-GFP or control AAV-GFP was injected into the hippocampus at both sides in 4-month-old 5 × FAD mice. Aβ deposition was analyzed by immunofluorescent staining one month after stereotaxic injection. Surprisingly, the number and the size of Aβ plaques were significantly reduced in the hippocampi of 5 × FAD mice expressing M compared to 5 × FAD mice expressing control GFP (Fig. S8a–e). However, the Aβ plaques in 5 × FAD mice expressing control GFP were large and loosely diffused while those in M-expressing mice were condensed (Fig. S8c, d). Quantitative evaluation of co-localization of microglia and Aβ showed significantly more Aβ deposits associated with microglia in hippocampi expressing M than that in hippocampi expressing control GFP (Fig. S8f, g). Consistent with increased neuronal loss in 5 × FAD mice expressing M, the 5 × FAD mice expressing M displayed decreased spontaneous alterations compared to 5 × FAD mice without M expression in the Y maze test (Fig. S8h). These results suggested that the dense plaques are more potent to activate microglia compared to diffused plaques, indicating that they are likely more toxic to neurons than diffused plaques. Together, M promotes formation of dense amyloid deposits, therefore increasing neuronal susceptibility to degeneration.

### Activation of microglia and astrocytes in mouse hippocampus with M expression

**Fig. 5:**
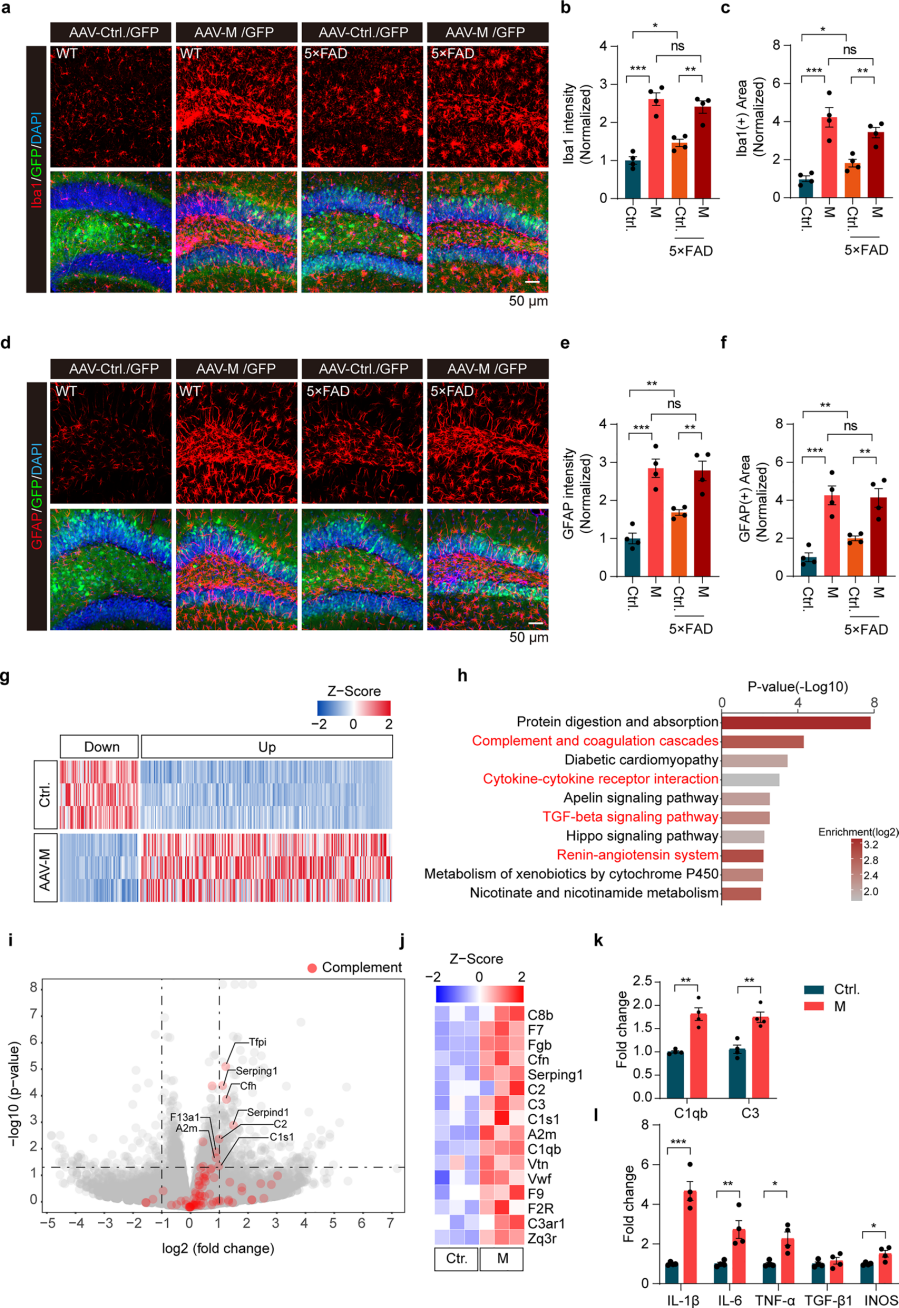
Activation of microglia and astrocytes with distinct transcriptional profiles in the hippocampus of WT and 5×FAD mice expressing M. **a-f** Confocal images of microglia (**a)** and astrocytes (**d)** in mouse hippocampus expressing either M or control. The intensity (**b**, **e**) and the area (**c**, **f**) of Iba1 or GFAP staining were measured and quantified, respectively. *P < 0.05, **P < 0.01, ***P < 0.001*.* Results were from three independent experiments, mean ± SEM, multi-way ANOVA followed by Dunnett’s test. **g** Heatmap of differentially expressed genes (DEGs) between mice expressing M and mice expressing control GFP (Ctrl.).* n* = 3 mice. **h** GO enrichment analysis of the upregulated genes in mouse hippocampus expressing M. The 10 most significantly enriched GO terms are presented with *P*-values (−Log10). **i** Volcano plot of the whole genome RNA-seq profile of the hippocampus expressing M. Genes with a threshold of twofold changes and adjusted *P*-values (< 0.05) are considered as significant. Data from three independent experiments, two-tailed moderated *t-*test. Red: genes related to the complement system. **j** Expression of the selected complement genes in the RNA-seq data derived from mouse hippocampus expressing M and control GFP (Ctrl). **k**, **l** Increased expression of C1qb, C3, IL-1β, IL-6, TNF-α, and INOS in mouse hippocampus expressing M determined by qPCR.* n* = 4 mice. *P < 0.05, **P < 0.01, ***P < 0.001. Mean ± SEM, unpaired Student’s *t-*test. ns, no significance

Chronic neuroinflammation can result from cell death, which in turn promotes neurodegeneration [46]. We next investigated neuroinflammatory responses after M expression. In the DG region expressing M, microglia exhibited an amoeboid shape with Iba1 staining, indicating profound activation (Fig. 5a). When control AAV-GFP was injected, the 5 × FAD mice showed slightly more activated microglia in the DG than the WT mice. However, there was no notable difference in microglial morphology in DG between 5 × FAD mice and WT mice expressing M (Fig. 5a–c). Similar activation patterns were observed for astrocytes (Fig. 5d–f). Together, expression of M leads to activation of microglia and astrocytes.

To understand the molecular changes induced by M, we further performed RNA sequencing (RNA-seq) using hippocampal samples from mice expressing M. DESeq analysis revealed a total of 341 DEGs (> 1.5 fold, FDR < 0.01), including 260 up-regulated and 81 down-regulated genes, in WT mice expressing M compared to controls (Fig. 5g). Gene ontology (GO) enrichment analysis of significantly upregulated genes showed that the top 10 function modules were related to complement cascades and cytokine-cytokine receptor interaction (Fig. 5h), consistent with activation of glia (Fig. 5a–f) and increased inflammation induced by M expression in mouse brain (Fig. 5h). In particular, expression of genes encoding complement molecules C2, C3, C1qb, and A2M was significantly increased (Fig. 5i, j). qPCR analysis further verified increased expression of complement-related genes *C1qb* and *C3* as well as pro-inflammatory genes *IL-6*, *IL-1β*, *TNF-α*, and *iNOS*, in the brains following M expression (Fig. 5k, l).

### Expression of SARS-CoV-2 M leads to behavioral and cognitive impairments in mice

**Fig. 6:**
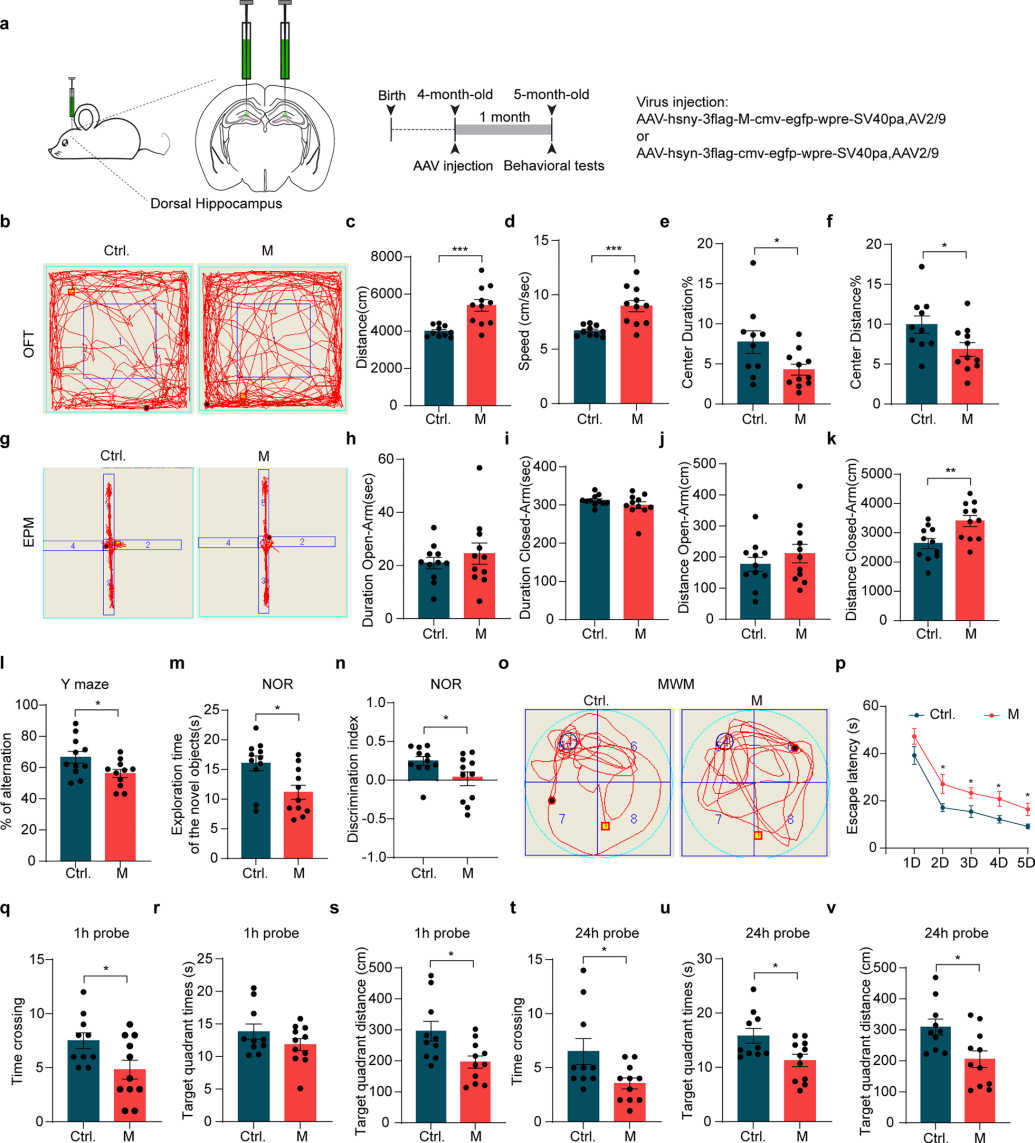
Expression of M leads to behavioral abnormality and cognitive impairments in WT mice. **a** Schematic illustration of AAV-mediated ectopic expression of M in mouse dorsal hippocampus. **b-f** Mouse performance in the open-field test. Representative traces (**b),** total distance traveled (**c)**, speed (**d)**, time spent in the center zone (**e**), and distance traveled in the center zone ( **f**) are shown. *P < 0.05, ***P < 0.001,* n* = 11. Mean ± SEM, unpaired Student’s *t-*test. **g–k** Mouse performance in the elevated plus maze test. Representative exploring traces (**g)**, time spent in the open (**h)** or closed arms (**i)**, and the total distance traveled through the open (**j)** or the closed arms (**k)** are shown. **P < 0.01*, n* = 11. Mean ± SEM, unpaired Student’s *t-*test. **l** The Y maze test. The percentage (%) of spontaneous alternation of mice expressing M or control (Ctrl.) was quantified. *P < 0.05*, n* = 11. Data are presented as mean ± SEM. Unpaired Student’s *t-*test. **m, n** Mouse performance in the novel-object recognition test. The exploration time of the new object (**m)** and the discrimination index (**n)** were quantified. *P < 0.05*, n* = 11. Mean ± SEM, unpaired Student’s *t-*test. **o-v** Mouse performance in the Morris water maze test (MWM). Representative traces (**o)** and escape latency during training period (1–5 days) (**p)** are shown. The times crossing the target quadrant (**q**, **t**), the time spent in the target quadrant (**r**, **u**), and distances traveled in the target quadrant (**s**, **v**) were quantified for the 1-h probe and 24-h probe trials, respectively.* n* = 10–11 mice/group. *P < 0.05*,* mean ± SEM, unpaired Student’s *t-*test

To examine functional impact of M expression in mice, behavioral analysis was performed (Fig. 6a). Open field test revealed significantly increased locomotor activity of mice expressing M, as evidenced by metrics including total distance traveled, speed, and the time and distance spent in the central zone (Fig. 6b–f). The elevated plus maze test showed no significant differences in the time and the distance spent in the open arm between control mice and mice expressing M. However, mice expressing M travelled longer distance than control mice (Fig. 6g–k). These results suggest that M expression in the hippocampus increases gross motor functions while having limited effects on anxiety-like behaviors. In the Y-maze test, mice expressing M showed a significant decrease in the percentage of effective alternation compared to control mice, indicating spatial working memory impairment (Fig. 6l). In the novel-object recognition test, mice expressing M also showed significant decreases in total time exploring the non-familiar object and in discrimination index compared to the control mice (Fig. 6m, n). The Morris water maze task showed that the control mice and the mice expressing M spent similar times to reach the visible platform, suggesting comparable locomotor function and swimming capacity of these mice. During the five days of hidden-platform training, mice expressing M showed a significantly longer escape latency on days 2, 3, 4, and 5 compared to control mice (Fig. 6o, p). Spatial memory was assessed at 1 and 24 h after the final training sessions. In the 1 h probe tests, mice expressing M demonstrated fewer crossings in the target quadrant (Fig. 6q) and reduced swimming distance (Fig. 6s) in the target quadrant compared to their control littermates. However, there was no significant difference in the time spent in the target quadrant between experimental and control mice (Fig. 6r). In the 24 h probe tests, mice expressing M exhibited fewer crossings in the target quadrant (Fig. 6t), less time in the target quadrant (Fig. 6u), and shorter swimming distance in the target quadrant compared to their control littermates (Fig. 6v). Collectively, these results suggest that expression of M in the hippocampus results in learning and memory impairment in mice.

### Expression of SARS-CoV-2 M disturbs the Golgi apparatus in *Drosophila*

To gain insight into the mechanism underlying mitochondrial defects induced by M, we performed RNAseq of the dissected fly muscles ectopically expressing M. A total of 214 DEGs (> twofold), including 147 up-regulated and 77 down-regulated genes, were identified in fly muscles expressing M compared to control muscles expressing mhc-gal4 alone (Fig. S9a). GO enrichment analysis of significantly up-regulated and down-regulated genes revealed that the top three function modules were protein refolding, defense response to Gram-positive bacterium, and mitochondrial complex I biogenesis (Fig. S9b, c). In muscles expressing M, expression of genes for protein refolding was upregulated while expression of genes for mitochondrial complex I biogenesis was downregulated (Fig. S9a–c).

**Fig. 7:**
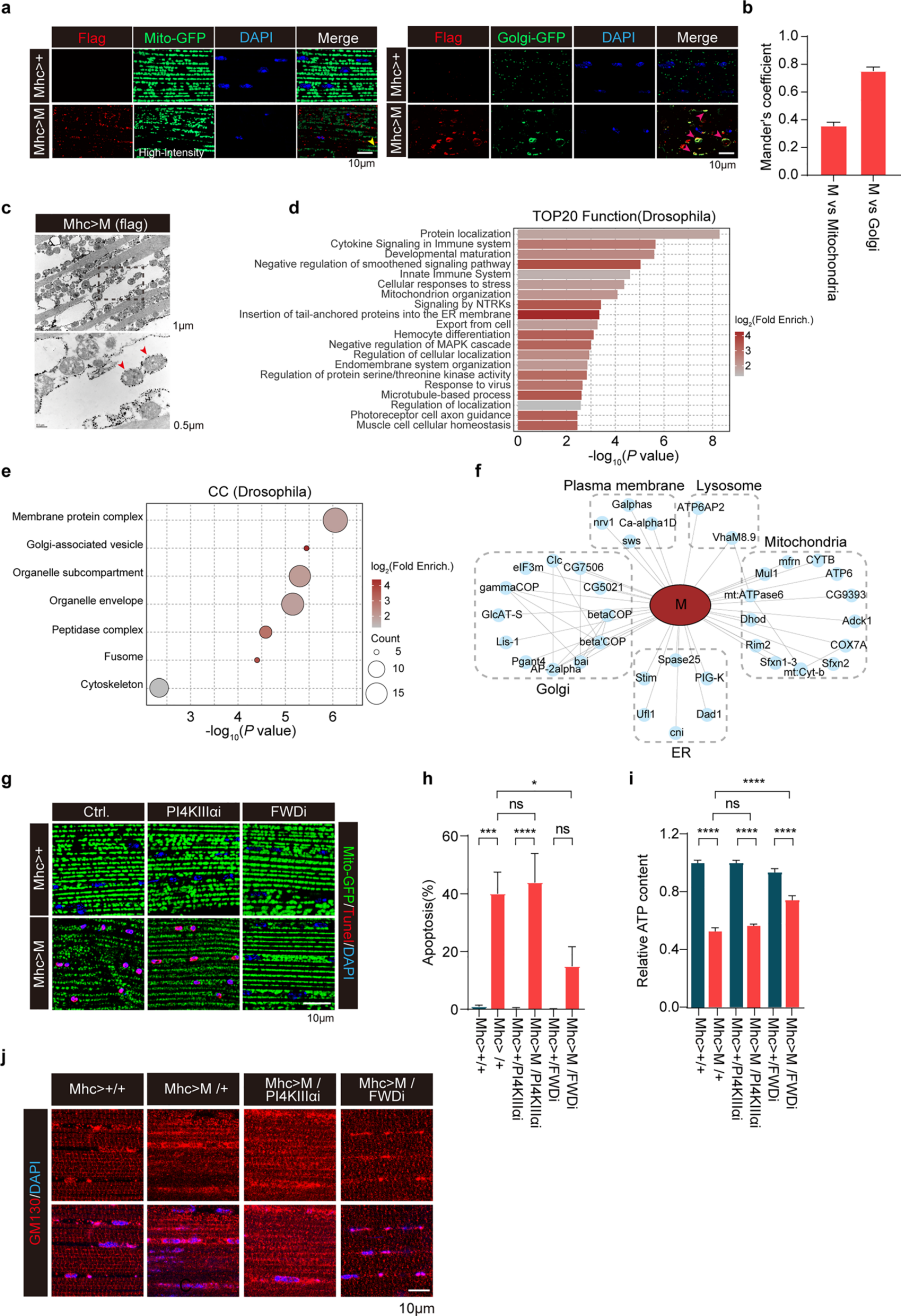
PI4KIIIβ knockdown restores the abnormal phenotypes and prevents cellular apoptosis induced by M in *Drosophila*. **a** Representative mitochondria (left) and Golgi’s complex (right) fluorescence images in indirect flight muscle (IFM) preparations from 3- to 5-day-old male flies expressing either Mhc-gal4 (Mhc > +) or Mhc-gal4-driven M (Mhc > M). Note that M was colocalized with Golgi and induced Golgi morphological abnormality. **b** Mander’s coefficient for colocalization of M with either mitochondria or Golgi. Three different regions from each IFM and > 6 flies for each experimental group were analyzed. **c** M in IFM of *Drosophila* was detected by an immune-gold labeled anti-flag antibody followed by TEM analysis. Red arrowheads: colocalization of M and mitochondria. **d**, **e** GO pathway analysis of M interactome in IFM tissues expressing M. Proteins enriched > 20-fold compared to control immunoprecipitation were included. The top 20 enriched GO biological functions (**d)** and enriched GO cellular components (**e)** are shown. Circle sizes scale to number of detected proteins. **f** Interactions between M (red ellipse) and *Drosophila* proteins (light-blue circles). Physical interactions among host proteins (thin black lines) were curated from STRING and visualized with Cytoscape. Results from 3 biologically independent samples were used. **g**, **h** FWD knockdown partially suppressed mitochondrial abnormality (**g)** and apoptosis (**h)** caused by M in IFM. Confocal microscopic sections of IFM from 3- to 5-day-old male flies expressing either mhc-gal4 (Mhc > +) or mhc-gal4-driven M (Mhc > M) followed by expressing either mhc-gal4 driven PI4KIIIα RNAi (Mhc >  + /PI4KIIIαi and Mhc > M/PI4KIIIαi, respectively), or mhc-gal4 driven FWD RNAi (Mhc >  + /FWDi and Mhc > M/FWDi). Apoptotic cell death was quantified. *P < 0.05, ***P < 0.001, ****P < 0.0001.* n* > 10 flies/genotype. Results were from three independent experiments, mean ± SEM, multi-way ANOVA followed by Dunnett’s test. **i** FWD RNAi significantly rescued the M-induced ATP reduction. ATP contents of thorax muscle tissues from the indicated genotypes were measured and normalized against the protein levels. ****P < 0.0001. Results were from three independent experiments, mean ± SEM, multi-way ANOVA followed by Dunnett’s test. **j** FWD knockdown reversed Golgi complex abnormalities induced by M in IFM. Golgi staining images of IFM tissues from 3- to 5-day-old male flies expressing mhc-gal4-driven M (Mhc > M) followed by expressing either mhc-gal4-driven PI4KIIIα RNAi (Mhc > M/PI4KIIIαi) or mhc-gal4-driven FWD RNAi (Mhc > M/FWDi) are shown. Flies expressing mhc-gal4 alone (Mhc >  + / +) or mhc-gal4-driven M alone (Mhc > M/ +) are included as controls. ns: no significance

M is one of the important functional components that plays a significant role in maintaining virion size and shape [47]. M is predicted to have three distinct transmembrane domains [47–49]. Studies have shown that M is localized on the Golgi membrane and mitochondrial out membrane in mammalian cell lines [50–52]. In this study, we employed two different fluorescent proteins, Mito-GFP (to label mitochondria) and Golgi-GFP (to label Golgi apparatus), to determine the subcellular localization of M in muscle cells in flies. Immunofluorescent staining showed that the M-positive vesicles were rarely colocalized with mito-GFP (Fig. 7a, left panels), but extensively colocalized with Golgi-GFP (Fig. 7a, right panels). Remarkably, we detected enlarged Golgi apparatus in muscle cells expressing M (Fig. 7a, right panels). Fewer mitochondrial localization of M in fly muscles was further verified by immunogold labeling followed by TEM analysis (Fig. 7c). Thus, M is mainly detected in and induces abnormality of the Golgi apparatus.

We next performed M interactome analysis using fly muscles expressing M. Fly muscles expressing mhc-gal4 alone were used as a control. Muscle lysates were immunoprecipitated with an anti-flag antibody followed by MS detection. A total of 118 M-interacting proteins were detected with > 20-fold changes in muscles expressing M compared to control muscles. GO enrichment analysis identified top 20 functional modules, including protein localization, innate immune system, cellular response to stress, and mitochondrial organization (Fig. 7d). Protein localization analysis revealed high enrichment of proteins from Golgi and mitochondria (Fig. 7e, f). Proteins involved in ER-Golgi transportation, particularly COP1-mediated retro-transport, were significantly enriched (*P* < 10^−5^, Fig. 7e). In HEK293 cells expressing M, M-interactome identified a total of 432 interacting proteins with > 20-fold enrichment compared with control cells transfected with empty plasmid. GO enrichment analysis of M-interacting proteins showed significant enrichment of proteins for Golgi vesicle transport, response to ER stress, and mitochondrion organization (Fig. S10a–c). Importantly, M interacts with ArfGEF1 and ArfGAP1, two regulators of Arf signaling, in Golgi (Fig. S10c). As the PI4KIIIβ/arf1 pathway participates in the crosstalk between Golgi complex and mitochondria [53], it is possible that M disrupts the Golgi complex, which in turn promotes mitochondrial defects.

### M induces mitochondrial fission and apoptosis via a PI4KIIIβ-mediated mechanism

Mitochondria are dynamic organelles that undergo continuous fusion and fission to maintain their morphology and functions. Mitofusins (Marf in *Drosophila*) and Drp1 are key components that regulate mitochondrial fusion and fission. We next expressed either Marf or Drp1 siRNA in fly muscles expressing M. Drp1 RNAi alone did not cause obvious mitochondrial fusion in control fly muscles. Consistent with a previous report [54], overexpression of Marf resulted in hyper-fused and damaged mitochondria (Fig. S11a, Morphology 4). In flies expressing M, both Marf overexpression and Drp1 RNAi partially suppressed mitochondrial fragmentation induced by M (Fig. S11a–c). This observation was further verified by TEM analysis (Fig. S9d). However, the inhibition of mitochondrial fragmentation did not restore the M-induced ATP reduction in *Drosophila* muscles (Fig. S11e), suggesting that fragmentation alone was not the cause of M-induced mitochondrial functional impairment. Consistent with ATP detection, neither Marf overexpression nor Drp1 RNAi suppressed apoptotic cell death induced by M in fly muscles (Fig. S11a, b, and f). Thus, mitochondrial dynamics abnormality is unlikely a cause of, but instead a phenotype accompanying, M-triggered cell death.

In addition to mitochondrial abnormality, we noticed that M expression disrupted Golgi apparatus (Fig. 7a). Recent studies suggest that the Golgi-derived PI(4)P-containing vesicles are key modulators of mitochondrial fission in both mammalian cells and *Drosophila* [53, 55]. We hypothesize that the M-induced mitochondrial fission was related to impairment of Golgi-derived PI(4)P-containing vesicles. Knockdown of four wheel drive (Fwd), a *Drosophila* homolog of mammalian PI4KIIIβ specific for generation of Golgi-derived PI(4)P-containing vesicles, did not change mitochondrial fission and apoptotic cell death in muscles of WT control flies. Remarkably, Fwd knockdown suppressed the M-induced Golgi disruption, mitochondrial fragmentation, reduced ATP content, and apoptotic cell death in *Drosophila* muscles (Fig. 7g–j). In contrast, knockdown of PI4KIIIα, a homolog of human PI4KIIIβ localized on plasma membrane, had little effect on M-induced Golgi disruption, mitochondrial fission and apoptotic cell death in *Drosophila* muscles. These results suggest that the Golgi-derived PI(4)P-containing vesicles are a critical regulator of M-induced Golgi disruption, mitochondria fragmentation, and cell death in *Drosophila* muscles. Thus, the Golgi abnormality induced by M is upstream of mitochondrial abnormalities and following cellular apoptosis in *Drosophila*.

### PI4KIIIβ inhibition alleviated neuronal perturbation caused by expression of SARS-CoV-2 M

**Fig. 8:**
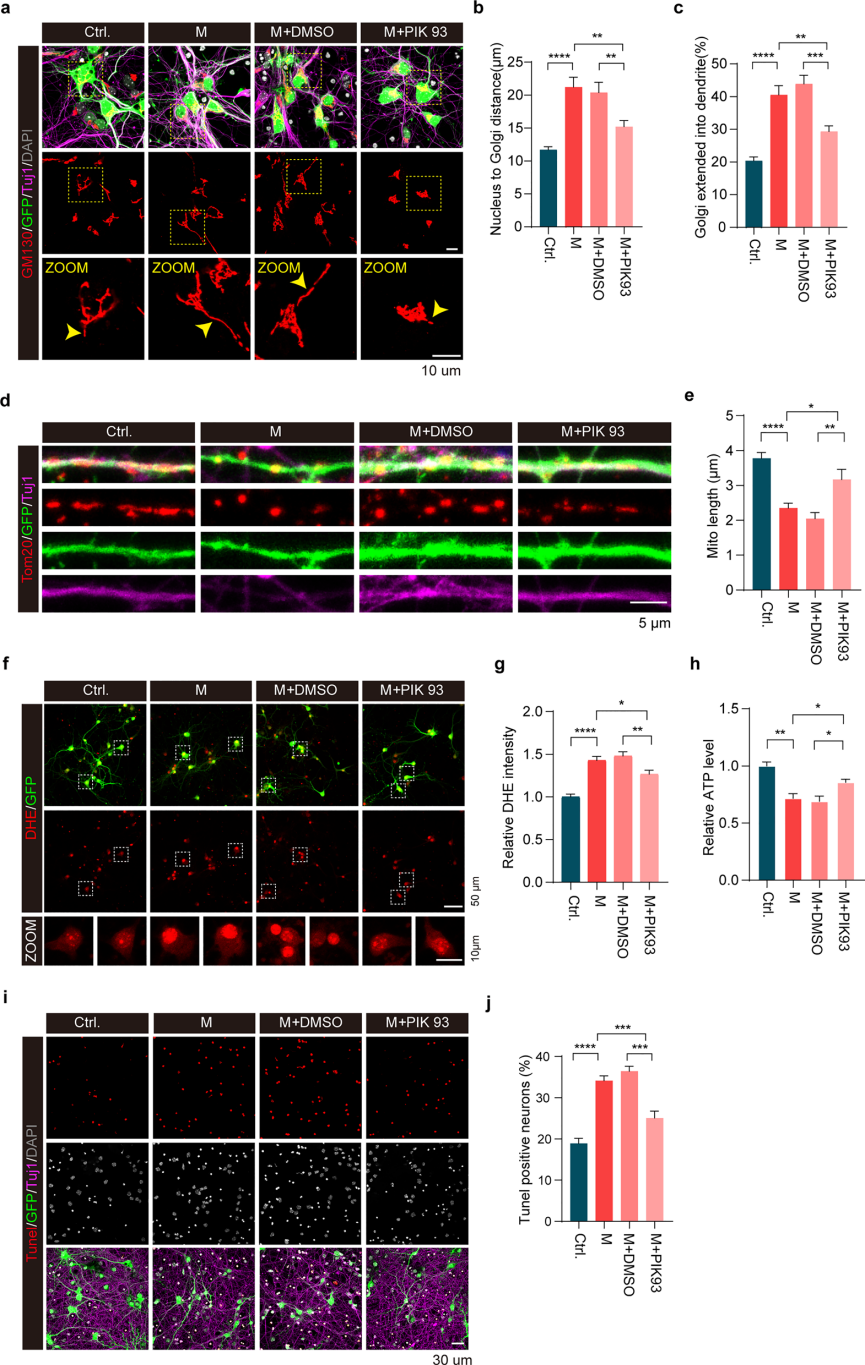
PI4KIIIβ inhibition rescues neuronal aberration caused by M expression. **a-c** Representative immunofluorescent staining for Golgi morphology in primary cortical neurons expressing either M or control (Ctrl) for 4 days followed by either PIK93 or vehicle (DMSO) treatment. Red: GM130 to label Golgi; Green: GFP to show AAV infection; Purple: Tuj1 to mark neurons. Yellow arrowheads indicate abnormal Golgi morphology. The distances from nucleus to Golgi (**b)** and the percentage of Golgi within dendrites (**c)** were measured and analyzed.* n* > 50 cells/ group. **P < 0.01, ***P < 0.001, ****P < 0.001*.* **d**, **e** Immunofluorescent staining for mitochondria in primary cortical neurons expressing either M or control (Ctrl) for 4 days followed by either PIK93 or vehicle (DMSO) treatment. Red: Tom20 to label mitochondria; Green: GFP to show AAV infection; Purple: Tuj1 to mark neuron. Mitochondrial length within dendrites was measured and analyzed (3–5 secondary dendrites per neuron and > 50 cells per indicated group). *P < 0.05, **P < 0.01, ****P < 0.0001. **f**, **g** DHE staining and neuronal relative DHE intensity.* n* > 100 cells/group. *P < 0.01, **P < 0.01, ****P < 0.0001*.* **h** ATP contents in neurons infected with AAV virus expressing either M or control (Ctrl.) followed by PIK93 treatment. *P < 0.01, **P < 0.01. **i**, **j** Tunel staining for neuronal apoptosis. Neuronal apoptosis (%) was analyzed.* n* > 100 cells/group. ***P < 0.001, ****P < 0.0001*.* All results were from three independent experiments, mean ± SEM, multi-way ANOVA followed by Dunnett’s test

We next analyzed Golgi structure in primary cultured mouse neurons after M expression. The Golgi complex was generally detected as tubular structures in the soma and in proximity to the nucleus in control neurons. However, neurons expressing M exhibited elongated Golgi tubules that extended into neuronal processes (Fig. 8a–c). Treatment with PIK-93, a known PI4KIIIβ inhibitor, significantly reversed neuronal defects induced by M expression, including the abnormal Golgi morphology, mitochondrial fragmentation and dysfunction, excessive ROS production, reduced ATP production and neuronal apoptosis (Fig. 8a–j). In contrast, PIK-93 had little effect on control neurons with no M expression (Fig. S12). These results suggest that M induces Golgi and mitochondrial abnormalities via PI4KIIIβ activation. Inhibition of PI4KIIIβ reverses Golgi and mitochondrial abnormality as well as neuronal degeneration caused by M.

## Discussion

Accumulating evidence indicates significant and long-lasting neurological manifestations of COVID-19 [11, 18, 40, 56]. About four out of five patients who sustained COVID-19 will show one or several neurological symptoms that can last months after the infection, ranging from loss of taste and smell, impaired concentration capability, fatigue, pain, sleep disorders, autonomic disorders and/or headache to psychological effects such as depression and psychosis [57]. Increased new diagnosis of neurodegenerative disorders after COVID-19 has also been reported [58]. Moreover, a pre-existing diagnosis of neurodegenerative diseases is associated with a subsequently increased risk of COVID-19, especially COVID-19-related death, indicating that COVID-19 may accelerate neurodegeneration in humans [59]. In this study, we demonstrated that expression of SARS-CoV-2 M caused hippocampal atrophy, neural apoptosis, glial cell activation, as well as Golgi and mitochondrial damage. Moreover, M was localized to the Golgi apparatus and genetically interacted with FWD (PI4KIIIβ) to regulate Golgi functions in flies. *Fwd* RNAi, but not PI4KIIIα RNAi, rescued the M-induced Golgi abnormalities, mitochondrial fragmentation, and ATP reduction. Inhibition of PI4KIIIβ activity suppressed the M-induced neuronal cell death. Furthermore, the M-induced mitochondrial fragmentation and apoptotic neuronal death were mediated by the Golgi-mitochondrial communication. To our knowledge, this study was the first to show that SARS-CoV-2 M induces neurodegeneration in mouse brains, providing a potential mechanism for long-lasting neurological symptoms of COVID-19. Remarkably, SARS-CoV-2 M accelerated neurodegeneration of 5 × FAD mice, which is consistent with clinical findings that COVID-19 advances neurodegeneration in humans. Particularly, the M-induced neurodegeneration in hippocampal formation is specific in DG and proximal CA3 regions. In contrast, CA1, CA2 and distal CA3 were not affected. Furthermore, this study identified disruption of PI(4)P-mediated Golgi-mitochondria interaction as a mechanism of M-induced neurodegeneration. Our findings suggest that inhibition of PI4KIIIβ is a potential therapeutic strategy for neurodegeneration associated with COVID-19.

A close correlation between COVID-19 and neurodegenerative disorders has been reported [58]. Brain atrophy has also been identified in individuals after infection [60]. Experiments showed that SARS-CoV-2 can directly infect both peripheral neurons and central neurons before viremia [61, 62]. However, there is a debate on whether COVID-19 neurological symptoms are caused by direct infection of neurons in the CNS or indirectly due to peripheral inflammatory factors. Results of this study demonstrate that SARS-CoV-2 M alone can induce neurological symptoms and neurodegeneration both in cultured neurons and in mouse brains. The M-induced neurodegeneration may be mediated by two mechanisms. One is increased activation of glial cells, particularly microglial cells. This is consistent with previous findings that microglia and astrocytes are activated following SARS-CoV-2 infection [63]. Another is that M induces Golgi abnormalities and therefore mitochondrial dysfunction in both *Drosophila* and mouse brains. Consistent with the notion, mitochondria in this study showed excessive fragmentation. TEM images further demonstrated fragmented mitochondria and severe damage of mitochondrial cristae. M expression induced significant reduction of mitochondrial membrane potential and ATP content, with markedly increased ROS production, suggesting mitochondrial dysfunction. It is well documented that mitochondrial dysfunction plays critical roles in neurodegeneration [37, 64, 65]. A recent study also suggests that M induces mitochondrial dysfunction, leading to abnormal calcium signaling [66]. Therefore, it is very likely that M induces neurodegeneration via a mechanism involving mitochondrial dysfunction.

Recent clinical studies suggest that older adults with COVID-19 are at a significantly increased risk of new diagnosis of AD [14]. Long-term cognitive decline is also common after SARS-CoV-2 infection [15]. Consistent with the clinical studies, expression of SARS-CoV-2 M is associated with significantly increased neurodegeneration in the hippocampus of WT and 5 × FAD mice. Hippocampus is one most affected brain region in patients with AD [67]. M-induced neurodegeneration in hippocampal formation is specific in DG and proximal CA3 regions. The rigid representations in aging result primarily from dysfunction of computational circuits involving DG and proximal CA3 [68]. Degeneration and dysfunction of the DG and proximal CA3 regions of the hippocampus play a significant role in memory and cognitive deficits characteristic of AD [69, 70]. Our results are consistent with clinical findings that older adults with COVID-19 are at a significantly increased risk for new diagnosis of AD [14]. A number of studies suggest that SARS-CoV-2 increases neuroinflammation including glial activation [71]. RNA-Seq results showed upregulation of inflammatory-related pathways, complement pathways, and microglial activation pathways in the hippocampus of 5 × FAD mice, suggesting roles of inflammation in M-induced neurodegeneration. M did not induce further activation of glial cells in 5 × FAD mice compared to that in normal control mice. It is possible that glial cell activation had reached a plateau level in 5 × FAD mice. Intriguingly, 5 × FAD mice expressing M exhibited smaller dense Aβ plaques than control 5 × FAD without M expression. Given the increased neuronal death in 5 × FAD mice expressing M, the dense Aβ plaques may be more toxic to neurons than the diffused Aβ plaques. Consistent with the notion, distinct strains of aggregates are proposed to be primary drivers of the phenotypic heterogeneity in AD. Particularly, dense plaques likely correlate with more severe toxicity and clinic symptoms [72–74]. The present study provides experimental evidence to support significantly increased risk of AD with COVID-19 infection.

In this study, we also found that M induced neuronal death via a PI4KIIIβ-mediated mechanism. During viral replication, viral structural proteins, including spike (S), E, M, and N, are translocated into ER membranes after translation, followed by transmission through the ER-to-Golgi intermediate compartment (ERGIC). In the ERGIC, N-encapsidated genome RNA interacts with structural proteins and buds into the lumen of secretory vesicular compartments. Finally, virions are secreted from infected cells by exocytosis [75]. During these processes, M directly interacts with ER and Golgi membranes and proteins for its transport and assembly. Therefore, M disturbs the Golgi function, resulting in mitochondrial fission during viral replication processes. Our finding of Golgi abnormality in cells expressing M supports this notion. M promotes Golgi-ER retro-transport, therefore increasing Golgi-derived PI(4)P-containing vesicles, leading to increased mitochondrial fission [53]. Knockdown of fwd (PI4KIIIβ), but not PI4KIIIα, significantly reversed the M-induced Golgi abnormality, mitochondrial fragmentation, and ATP reduction. Remarkably, PI4KIIIβ inhibitor PIK93 reverses M-induced Golgi abnormality, mitochondrial dysfunction and neuronal death [76]. Thus, the Golgi-derived PI(4)P-containing vesicles contribute to mitochondrial fission, resulting in accumulation of fragmented and damaged mitochondria, leading to reduced ATP production, increased ROS production, and eventually cell death.

In summary, this study reveals a novel, M-mediated mechanism of action of SARS-CoV-2 in brains. Our results open a new avenue for treatment of COVID-19-associated neurodegeneration.

## Conclusions

In summary, this study shows that expression of SARS-CoV-2 M causes hippocampal atrophy, neuronal apoptosis, glial cell activation, Golgi apparatus and mitochondrial damage. M induces mitochondrial fragmentation and neuronal death via disruption of PI(4)P-mediated Golgi-mitochondrial communication. These findings provide a potential mechanism for long-lasting neurological symptoms of COVID-19 infection and suggest that inhibition of PI4KIIIβ is a potential strategy to treat neurodegeneration associated with COVID-19.

## Supplementary Information

 **Additional file 1**. **Figure S1**. Expression of SARS-CoV-2 structural and accessory proteins in *Drosophila*. **Figure S2**. Cell death and mitochondrial damage in *Drosophila* muscles caused by M expression show sex differences. **Figure S3**. p35 and DIAP1 inhibits SARS-CoV-2 M induced cell death in *Drosophila*. **Figure S4**. Expression of SARS-CoV-2 N does not induce neuronal cell death in primary neuron. **Figure S5**. TEM analysis of lung and turbinate tissues of minks infected with SARS-CoV-2 virus. **Figure S6**. Expression of either SARS-CoV-2 M or orf6 impairs mitochondria in muscle tissue. **Figure S7**. Expression of SARS-CoV-2 N does not induce neurodegeneration in hippocampus, unlike M. **Figure S8**. Expression of SARS-CoV-2 M facilitates Aβ plaque compaction in 5×FAD mice. **Figure S9**. RNAseq analysis of the dissected fly muscles that specifically expressed M. **Figure S10**. SARS-CoV-2 M interactome in HEK293 cells. **Figure S11**. Inhibition of mitochondrial fragmentation unlikely restores mitochondrial function impaired by M. **Figure S12**. PI4KIIIβ inhibitor PIK-93 treatment has little impact on normal control neurons.

 **Additional file 2**. **Table S1**. Antibody resource

## Data Availability

All data are available in the main text or supplementary materials. The datasets generated during and/or analyzed during the current study are available from the corresponding author on reasonable request.

## Abbreviations

COVID-19: Coronavirus disease 2019
SARS-CoV-2: Severe acute respiratory syndrome coronavirus 2
M: Membrane protein
E: Envelope protein
N: Nucleocapsid protein
AD: Alzheimer’s disease
IFM: Indirect flight muscles
AAV: Adeno-associated virus
Aβ: Amyloid-β
FWD: Four wheel drive (*Drosophila* phosphatidylinositol 4-kinase IIIβ homologue)
PI(4)P: Phosphatidylinositol 4-phosphate
