# Supplementary material for: SARS-CoV-2 membrane protein induces neurodegeneration via affecting Golgi-mitochondria interaction
Source: Transl Neurodegener. 2024 Dec 27;13:68. doi: 10.1186/s40035-024-00458-1 (PMC11674522; doi:10.1186/s40035-024-00458-1)
Supplement: Supplementary file 1 — Additional file 1. Figure S1. Expression of SARS-CoV-2 structural and accessory proteins in Drosophila. Figure S2. Cell death and mitochondrial damage in Drosophila muscles caused by M expression show sex differences. Figure S3. p35 and DIAP1 inhibits SARS-CoV-2 M induced cell death in Drosophila. Figure S4. Expression of SARS-CoV-2 N does not induce neuronal cell death in primary neuron. Figure S5. TEM analysis of lung and turbinate tissues of minks infected with SARS-CoV-2 virus. Figure S6. Expression of either SARS-CoV-2 M or orf6 impairs mitochondria in muscle tissue. Figure S7. Expression of SARS-CoV-2 N does not induce neurodegeneration in hippocampus, unlike M. Figure S8. Expression of SARS-CoV-2 M facilitates Aβ plaque compaction in 5×FAD mice. Figure S9. RNAseq analysis of the dissected fly muscles that specifically expressed M. Figure S10. SARS-CoV-2 M interactome in HEK293 cells. Figure S11. Inhibition of mitochondrial fragmentation unlikely restores mitochondrial function impaired by M. Figure S12. PI4KIIIβ inhibitor PIK-93 treatment has little impact on normal control neurons. [file 40035_2024_458_MOESM1_ESM.docx]

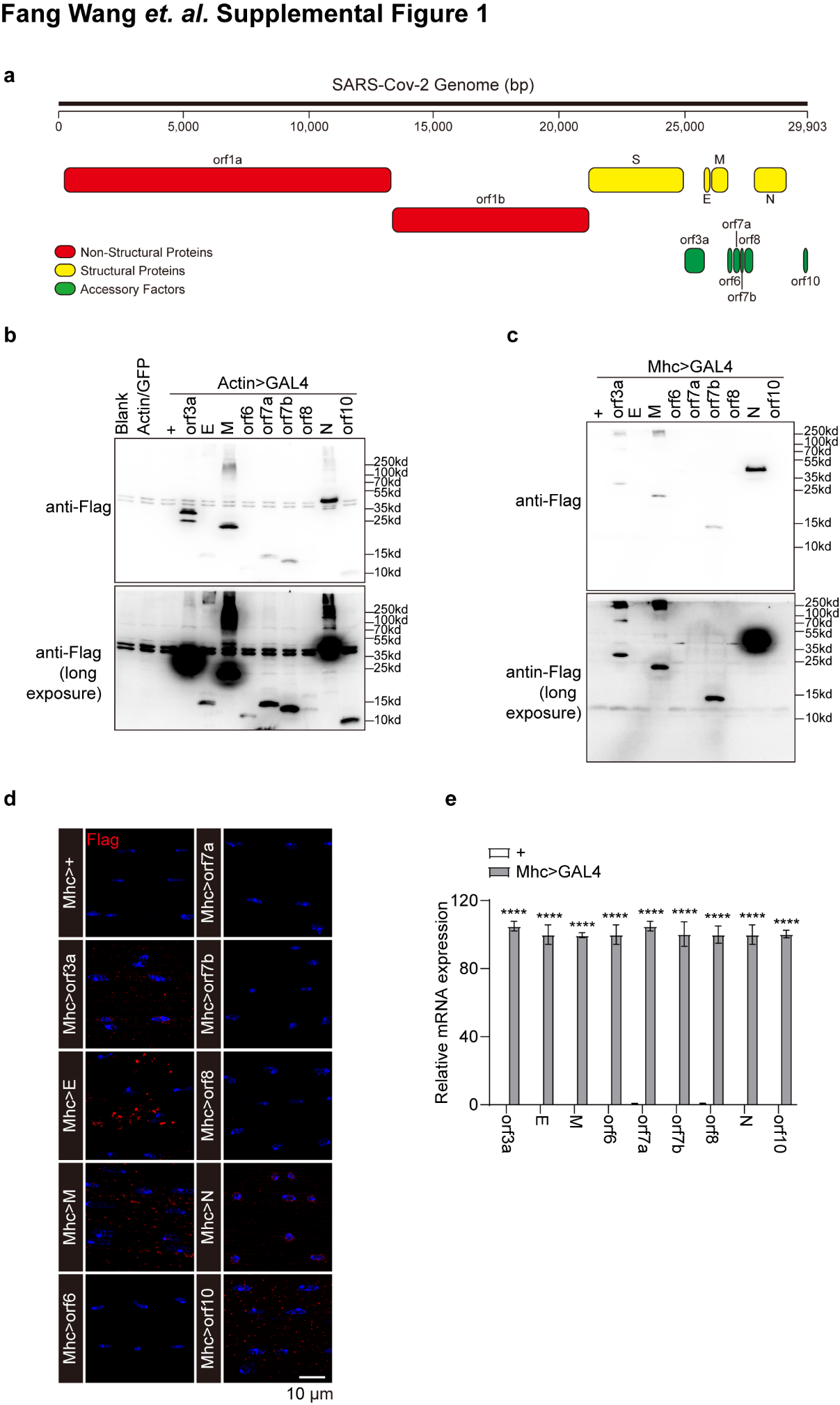


**Supplemental Figure 1. Expression of SARS-CoV-2 structural and accessory proteins in *Drosophila*.**

**a** Schematic presentation of the SARS-CoV-2 genome annotation and the canonical subgenomic mRNAs. The 29903bp RNA genome was translated to orf1a, orf1b, four structural proteins (S, E, M, and N), and at least six accessory proteins (orf3a, orf6, orf7a, orf7b, orf8, and orf10).

**b, c** Immunoblotting detection of flag-tagged SARS-CoV-2 proteins. Expression of SARS-CoV-2 proteins, including orf3a, E, M, orf6, orf7a, orf7b, orf8, N, or orf10, in S2 cells driven by actin-gal4 (**b**) and indirect flight muscle driven by mhc-gal4 (**c**) are shown.

**d** Representative immunostaining images of SARS-CoV-2 proteins in indirect flight muscle of *Drosophila*. Expression of SARS-CoV-2 proteins, including orf3a, E, M, orf6, orf7a, orf7b, orf8, N, or orf10, in indirect flight muscle was detected by immunofluorescent staining using an anti-flag antibody. Red: anti-flag staining, to label SARS-CoV-2 proteins; Blue, DAPI staining, to detect nuclear DNA. Bar = 10 μm.

**e** Relative mRNA levels of SARS-CoV-2 genes in indirect flight muscles of *Drosophila*. mRNA levels of SARS-CoV-2 genes were measured by real-time quantitative PCR. *****p <* 0.0001. Results from three independent experiments are expressed as mean ± SEM. The statistical analysis: unpaired Student's t-test.


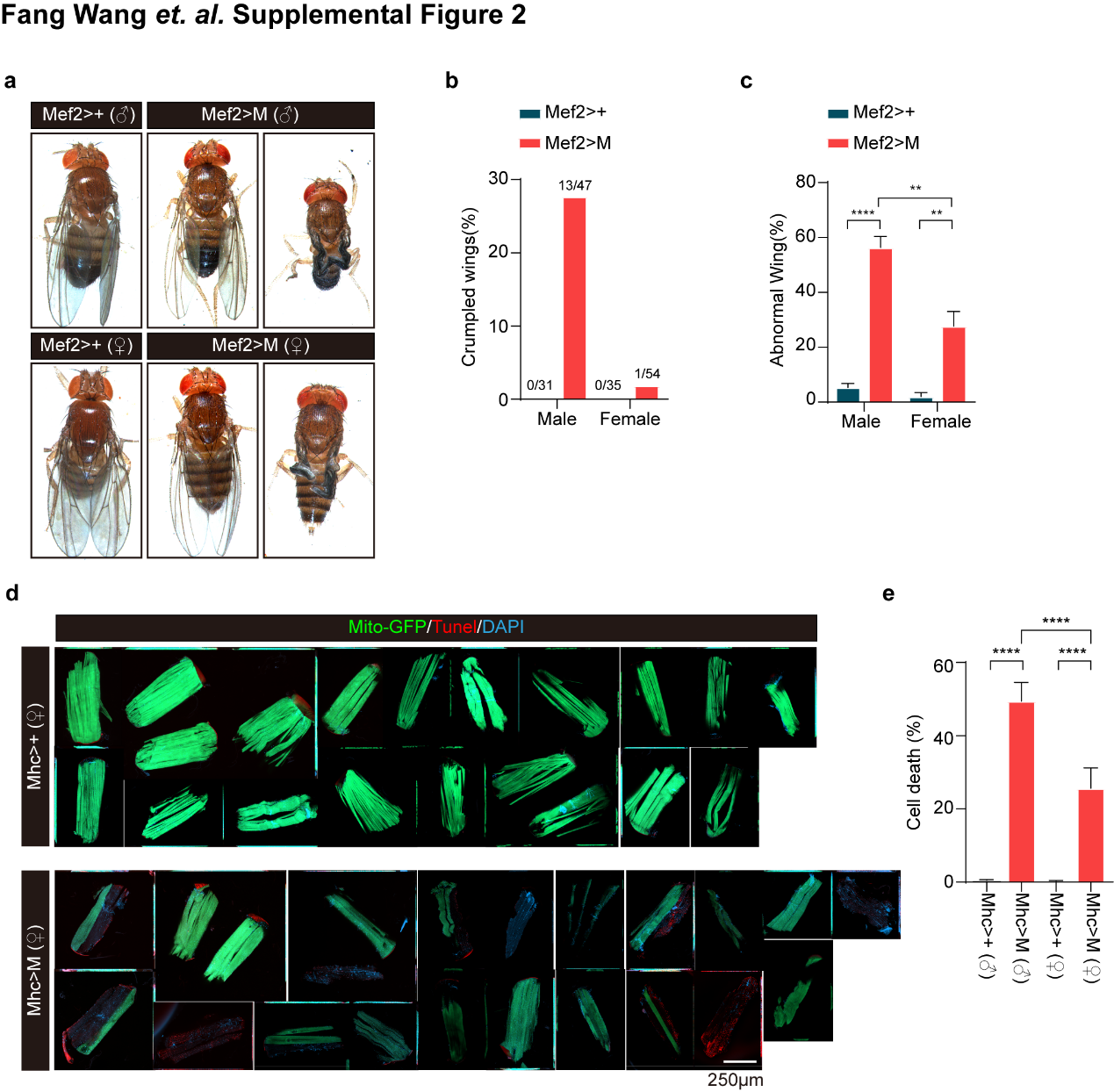


**Supplemental Figure 2. Cell death and mitochondrial damage in *Drosophila* muscles caused by M expression show sex differences.**

**a-c** Wing phenotypes of flies expressing SARS-CoV-2 M driven by mef2-gal4 (Mef2>M) were analyzed. Flies expressing mef2-gal4 alone (Mef2>+) were included as control. Crumbled wing phenotype was observed in flies expressing M (**a**). Crumbled wings (%) were quantified in both male and female flies, the numbers of indicated subtypes are counted and shown in the statistical chart (**b**). Abnormal wings (%) were quantified in both male and female flies and analyzed (**c**). ***p* < 0.01, *****p <* 0.0001. n > 50 flies/group. Results are expressed as mean ± SEM. The statistical analysis: Multi-way ANOVA followed by Dunnett’s test.

**d, e** Representative Tunel staining images of IFM tissues from 3- to 5-day-old Female (♀) flies expressing either Mhc-gal4 (Mhc>+) or Mhc-gal4 driven SARS-CoV-2 M (Mhc>M) are shown (**d**). Each piece IFM was dissected from one fly. Green: Mito-GFP; Red: Tunel staining positive; Blue: DAPI staining. Bar = 250 μm. Cell death (%) in each genotype flies were quantified, n > 20 flies/group were analyzed (**e**). *****p < 0.0001.* Results are expressed as mean ± SEM. The statistical analysis: Multi-way ANOVA followed by Dunnett’s test.


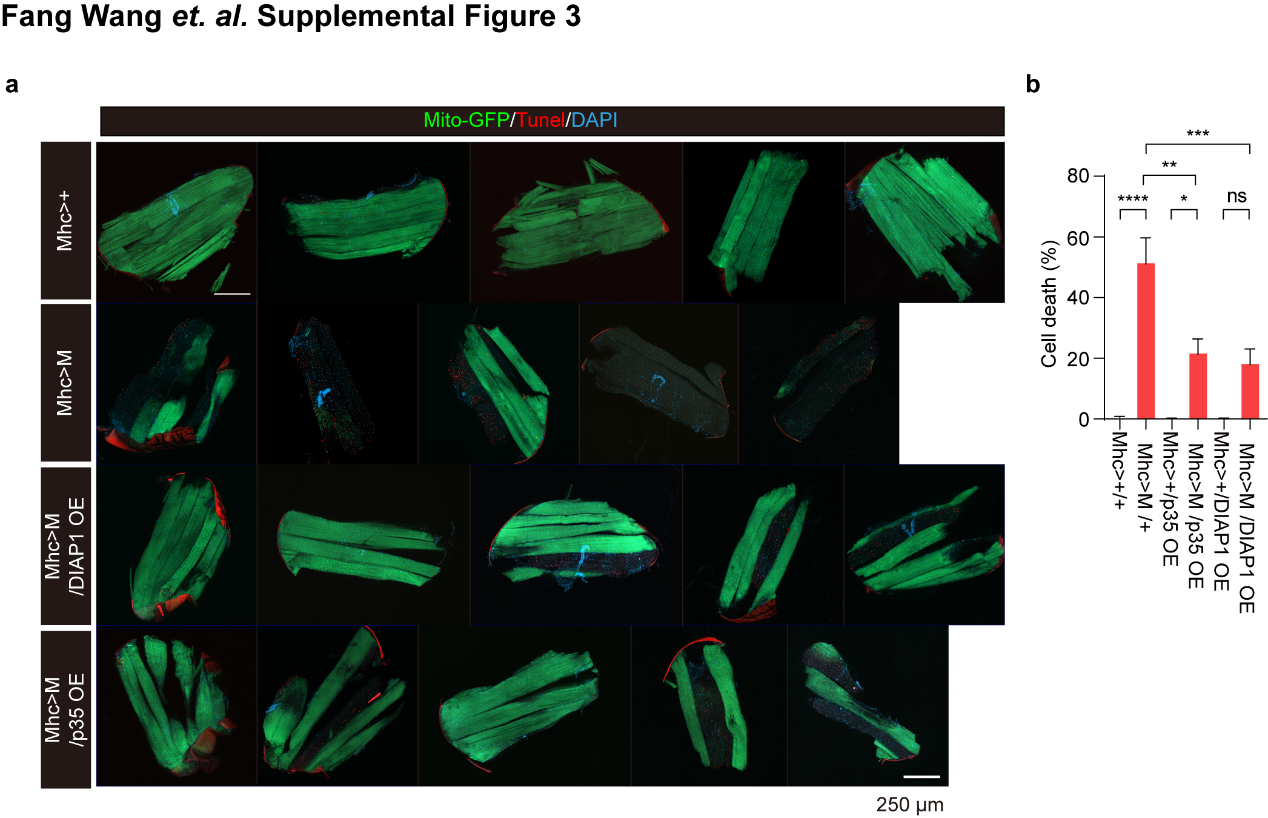


**Supplemental Figure 3. p35 and DIAP1 inhibits SARS-CoV-2 M induced cell death in *Drosophila*.**

**a** Representative Tunel staining images of indirect flight muscle sections from 3- to 5-day male flies expressing either mhc-gal4 alone (Mhc>+), mhc-gal4 driven M (Mhc>M), mhc-gal4 driven M and DIAP1 (Mhc>M/DIAP1 OE), or mhc-gal4 driven M and p35 (Mhc>M/p35 OE). Green: Mito-GFP, to detect mitochondria; Red: Tunel staining, to detect apoptotic cell death; Blue: DAPI staining, to detect nuclear DNA. Bar = 250 μm.

**b** Quantitative analysis of M-induced cell death in the presence of p35 or DIAP1. Expression of proteins was driven by mhc-gla4. Mhc>+/+: indirect flight muscle expressing mhc-gal4 alone. Mhc>M/+: indirect flight muscle expressing M. Mhc>+/p35 OE: indirect flight muscle expressing p35 alone. Mhc>M/p35 OE: indirect flight muscle expressing both M and *Drosophila* p35. Mhc>+/DIAP1 OE: indirect flight muscle expressing *Drosophila* DIAP1. Mhc>M/DIAP1 OE: indirect flight muscle expressing both M and Drosophila DIAP1. **p <* 0.05, ***p <* 0.01, ****p <* 0.001, *****p <* 0.0001, *ns:* no significance*.* n >10 flies/group. Results are expressed as mean ± SEM. The statistical analysis: Multi-way ANOVA followed by Dunnett’s test.


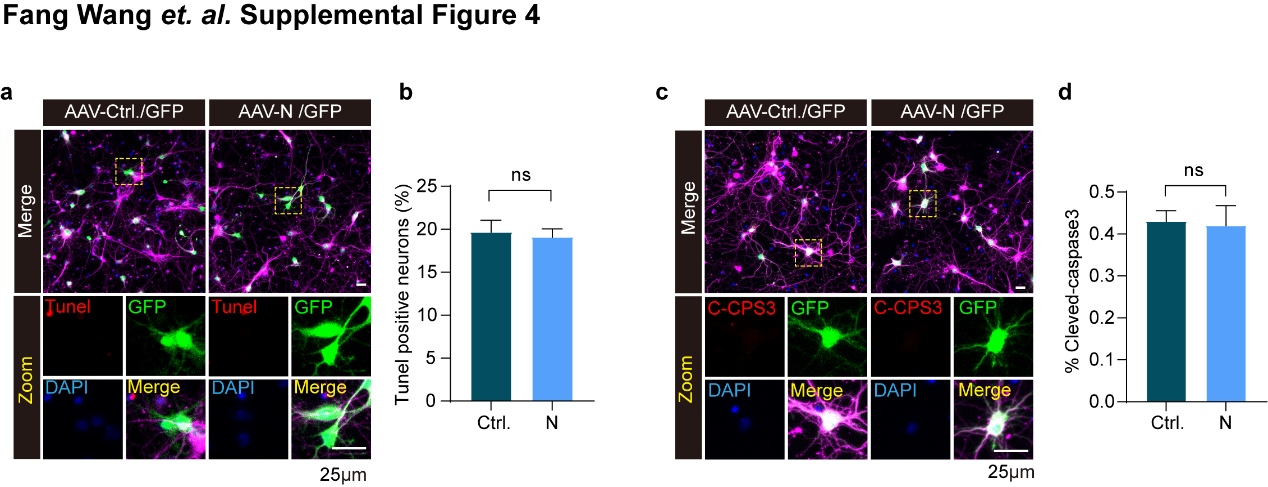


**Supplemental Figure 4.** **Expression of SARS-CoV-2 N does not induce neuronal cell death in primary neuron.**

**a-d** Apoptotic cell death analysis in primary neuronal cultures expressing N. Tunel and immunofluorescent staining of cleaved caspase-3 were performed for primary cortical neurons infected with AAV virus expressing either N or control (Ctrl.) for 4 days. Representative images of Tunel (**a**) and Cleaved-caspase 3 (C-CPS3) staining (**c**) of infected neurons are shown. Enlarged images are shown in the lower panels (Zoom). Red: Tunel staining positive (**a**) and C-CPS3 staining (**c**); green: GFP; blue: DAPI. Bar=25 μm. The percentage (%) of red positive neurons/total neurons were analyzed (**b**, **d**), n > 100 neurons/group. *ns* = *no significance.* Results from three independent experiments are expressed as mean ± SEM. The statistical analysis: unpaired Student's t-test.


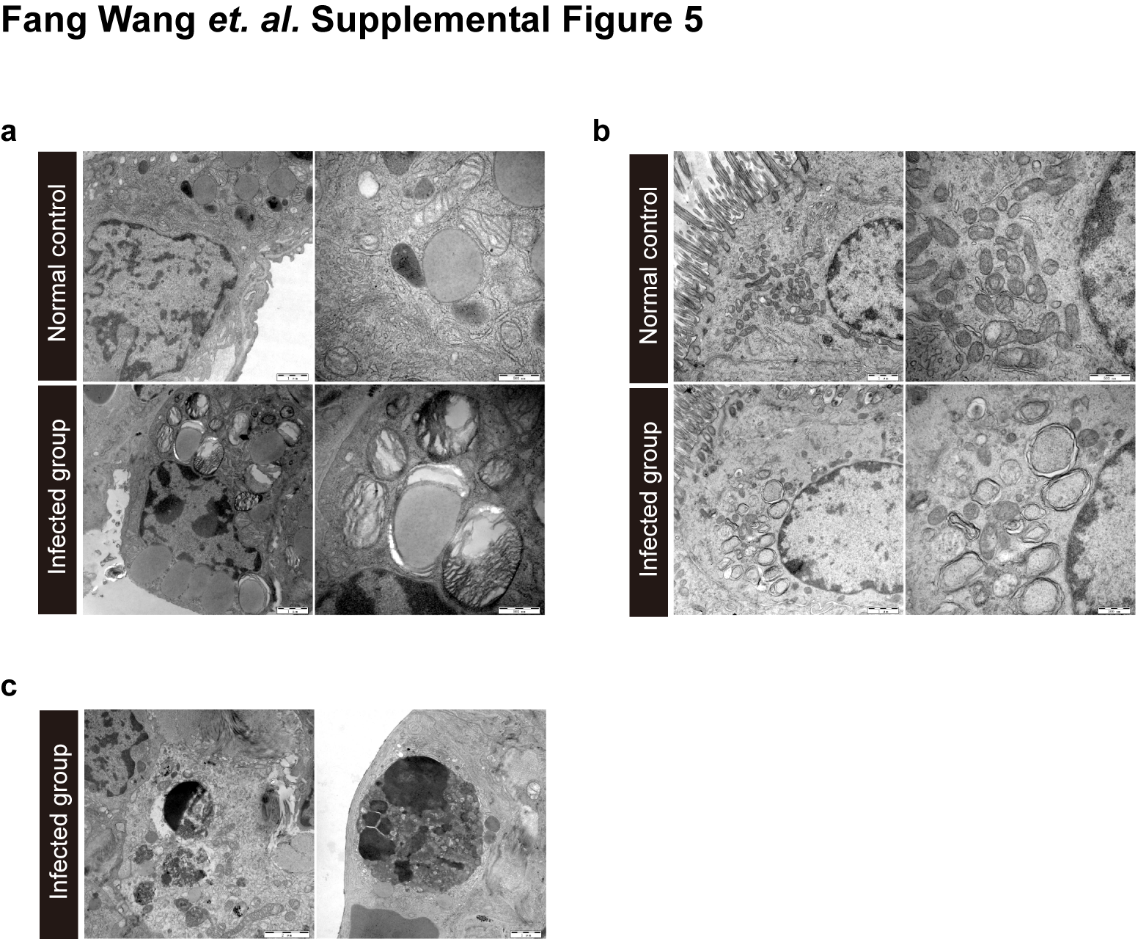


**Supplemental Figure 5. TEM analysis of lung and turbinate tissues of minks infected with SARS-CoV-2 virus.**

**a-c** Minks were infected with SARS-CoV-2 virus or mock infected with PBS (as a control) for 4 days. TEM images from lungs (**a**) and nasal turbinates (**b**) were shown. In each panel, normal control (upper) and infected group (lower) are included. Bar = 2 μm. Magnified images were shown in each panel (right). Bar = 1 μm. Images of apoptotic body (left) and apoptotic bodies engulfed by macrophage (right) in lung tissue of infected minks were shown (**c**). Bar = 2 μm (Left panels) and 1 μm (Right panels).


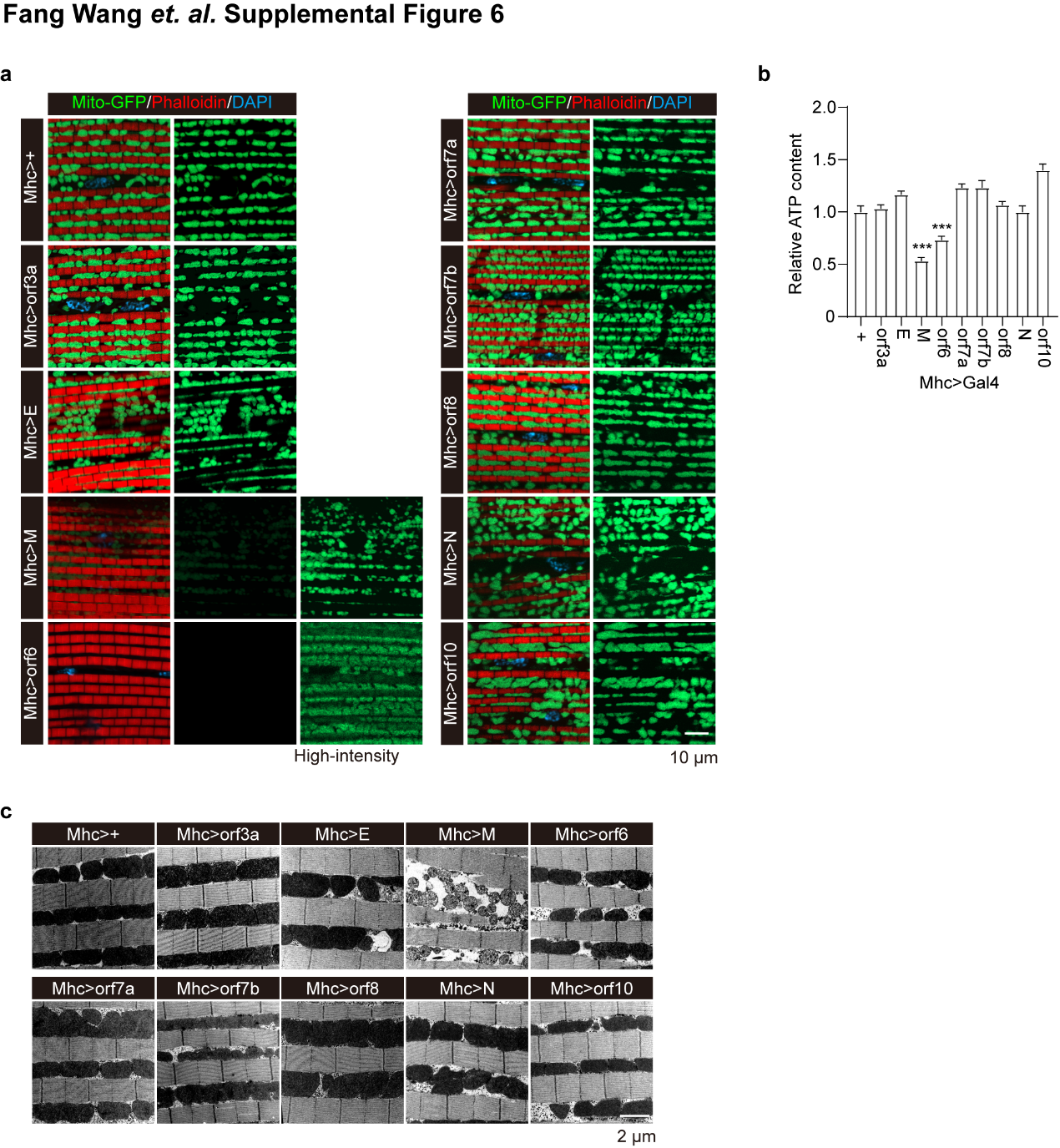


**Supplemental Figure 6.** **Expression of either SARS-CoV-2 M or orf6 impairs mitochondria in muscle tissue.**

**a** Representative images of Mito-GFP labeled mitochondria in indirect flight muscle from 3-5 days male flies expressing either mhc-gal4 (Mhc>+) (as a control) or Mhc-gal4 driven SARS-COV-2 related genes (orf3a, E, M, orf6, orf7a, orf7b, orf8, N or orf10) are shown. Mito-GFP: green, to detect mitochondria; Phalloidin: red, to detect myofilament; DAPI: blue, to detect nucleus. Bar = 10 μm.

**b** ATP contents of thorax muscle tissues from the indicated genotypes were measured and normalized against the protein levels. ****p<0.001*. Data was presented as mean ± SEM. Statistical analysis: One-way ANOVA followed by Tukey’s test.

**c** Representative TEM images of indirect flight muscle from 3-5 days male flies expressing either mhc-gal4 (Mhc/+) (as a control) or expressing Mhc-gal4 driven SARS-COV-2 related genes (orf3a, E, M, orf6, orf7a, orf7b, orf8, N, or orf10) are shown. Bar = 2 μm.


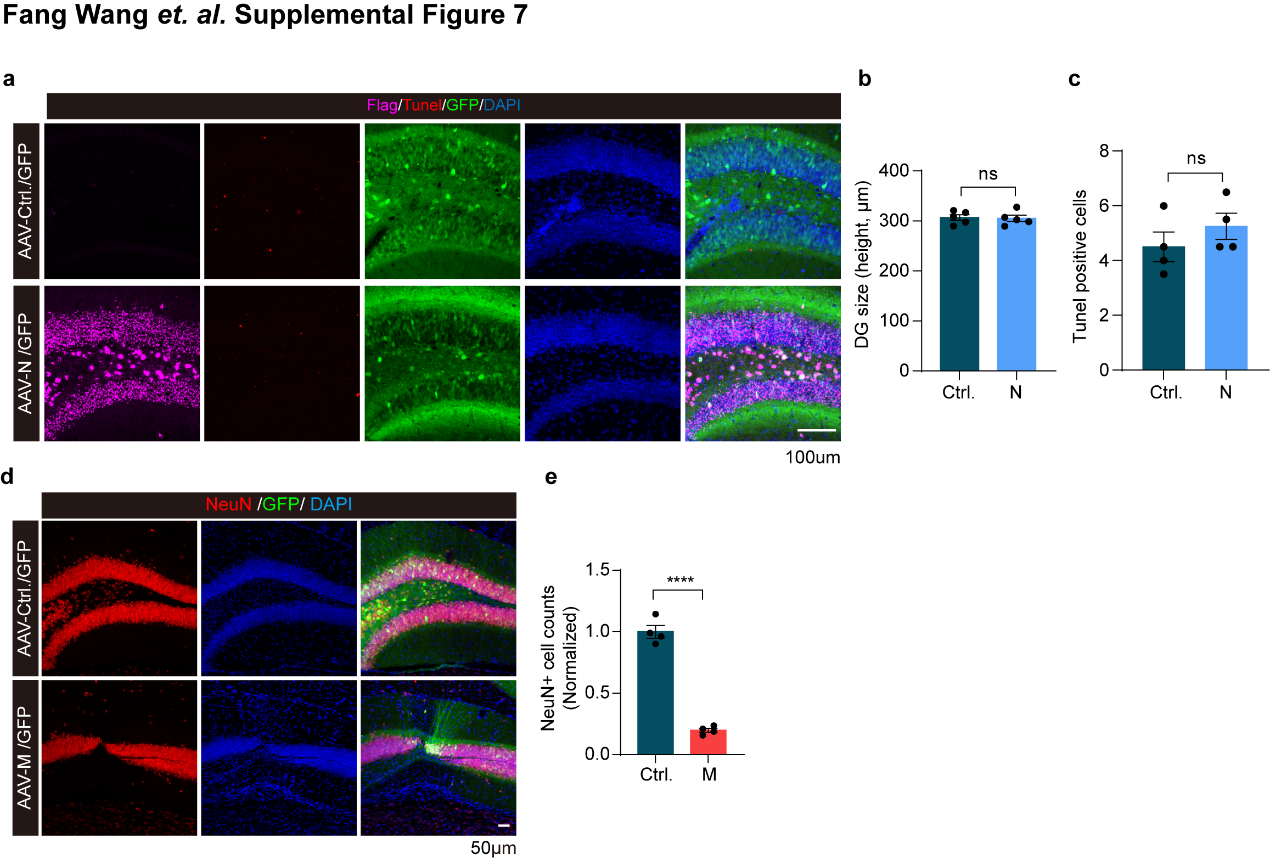


**Supplemental Figure 7.** **Expression of SARS-CoV-2 N does not induce neurodegeneration in hippocampus, unlike M.**

**a-c** Immuno-staining to detect cell death in mouse hippocampus expressing N. Representative immunofluorescence images of DG are shown (**a**). Red: Tunel assay signal to detect cell death; Purple: FLAG to label N protein expression; Green: GFP to detect AAV infection; Blue: DAPI to label nuclei. Bar = 100 μm. The height of Dorsal DG (**b**) and the number of Tunel-positive neurons (**c**) in hippocampus were quantified, respectively. n = 4 mice per indicated group. ns = no significance. Results from three independent experiments are shown as mean ± SEM. The statistical analysis: unpaired Student's t-test.

**d, e** M induces region-specific neurodegeneration in DG of mouse brain. Immunostaining of hippocampus after expressing either Mpro (left brain) or control GFP (right brain, Ctrl.) for 4 months (**d**). Red: NeuN to detect neuron; Green: GFP to show AAV infection; Blue: DAPI to stain nuclei. Bar = 50 μm. The number of neurons in Dorsal DG was quantified (**e**). *****p < 0.0001.* Results from three independent experiments are shown as mean ± SEM. The statistical analysis: unpaired Student's t-test.


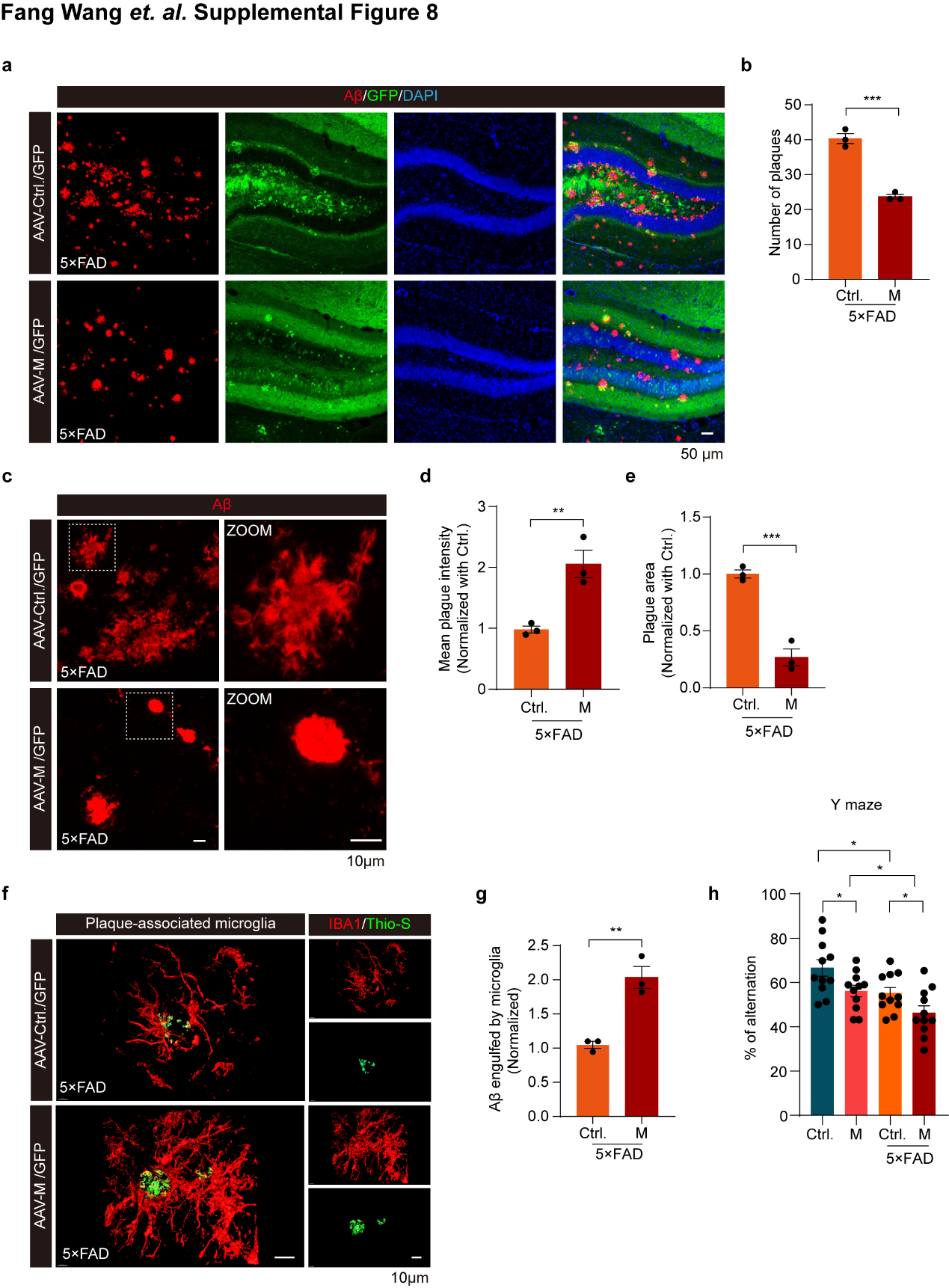


**Supplemental Figure 8. Expression of SARS-CoV-2 M facilitates Aβ plaque compaction in 5×FAD mice.**

**a, b** Aβ staining of 5×FAD mouse hippocampi expressing either M (left brain) or control GFP (right brain, Ctrl) are presented. Representative images of Aβ staining are shown (**a**). Aβ (red): to label plaques; AAV (GFP, green): to detect M expression; DAPI (blue): to detect nuclei. Bar = 50 μm. The number of plaques in each subgroup was quantified (**b**). 3-5 fields per mouse in each indicated group, n = 3 mice/group (**b**). ****p <* 0.001*.* Results are expressed as mean ± SEM. The statistical analysis: unpaired Student's t-test.

**c-e** Amplified images of Aβ in 5×FAD mouse hippocampi expressing either M (left brain) or control GFP (right brain, Ctrl.) are presented. Representative staining images are shown (**c**). Aβ (red): to label plaques. Bar = 10 μm. The mean plaque intensity (**d**) and plaque area (**e**) are quantified, 3-5 fields per mouse in each indicated group, n = 3 mice/group. ***p <* 0.01, ****p <* 0.001*.* Results are expressed as mean ± SEM. The statistical analysis: unpaired Student's t-test.

**f, g** A three-dimensional reconstruction of microglia and fibrillar Aβ. Representative staining images are shown (**f**). IBA1 (red): to label microglia; ThioS (green): to label plaques. Bar = 10 μm. The localization of Aβ in microglia is quantified (**g**), 8-12 fields per mouse in each indicated group. n = 3 mice/group (**g**). ***p <* 0.01*.* Results are expressed as mean ± SEM. The statistical analysis: unpaired Student's t-test.

**h** The Y maze test. The percentage (%) of spontaneous alternation of 5×FAD mice and WT with expressing M or control GFP (Ctrl.) was quantified. **p < 0.05*, n = 11. Data was presented as mean ± SEM. The statistical analysis: Multi-way ANOVA followed by Dunnett’s test.


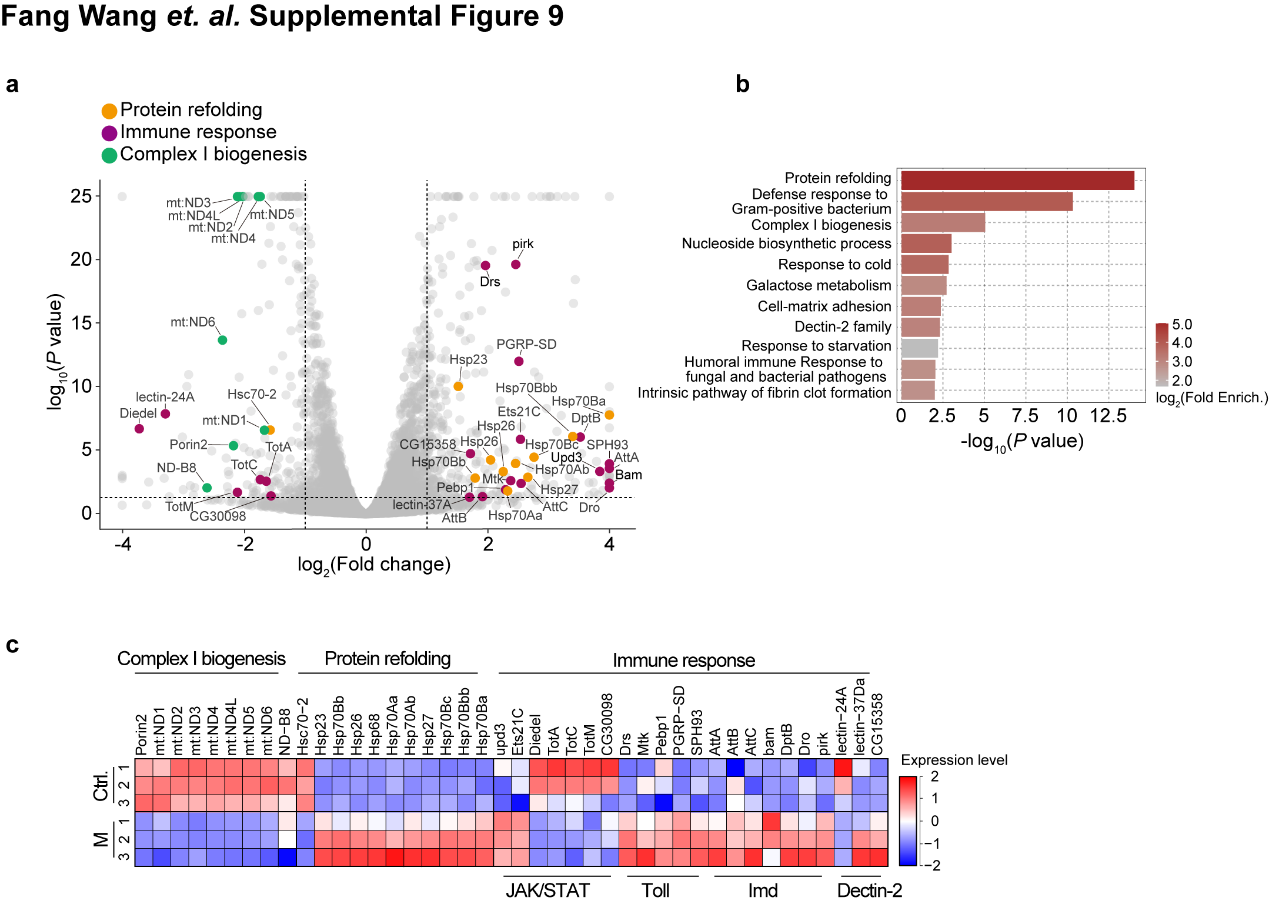


**Supplemental Figure 9. RNAseq analysis of the dissected fly muscles that specifically expressed M.**

**a** Volcano plot of the RNA-seq profile in IFM tissues from 3-5 days male flies expressing either mhc-gal4 (Mhc>+) or Mhc-gal4 driven SARS-CoV-2 Membrane protein (Mhc>M) shows genes with their threshold 2-fold changes and adjusted p-values to consider as significant genes. Data is from three independent experiments. Adjusted P value: two-tailed moderated t-test. Yellow: Refolding related genes; purple: immune response-related genes; green: mitochondrial Complex I-related genes.

**b** GO pathway analysis for the RNA-seq profile shown in (**a**), and the top 10 enriched GO biological functions for the 2-fold changed gene expression are shown.

**c** Heat map of the log fold changes for the genes indicated in (**a**) between Mhc>+ and Mhc>M group. Data is from three independent experiments.


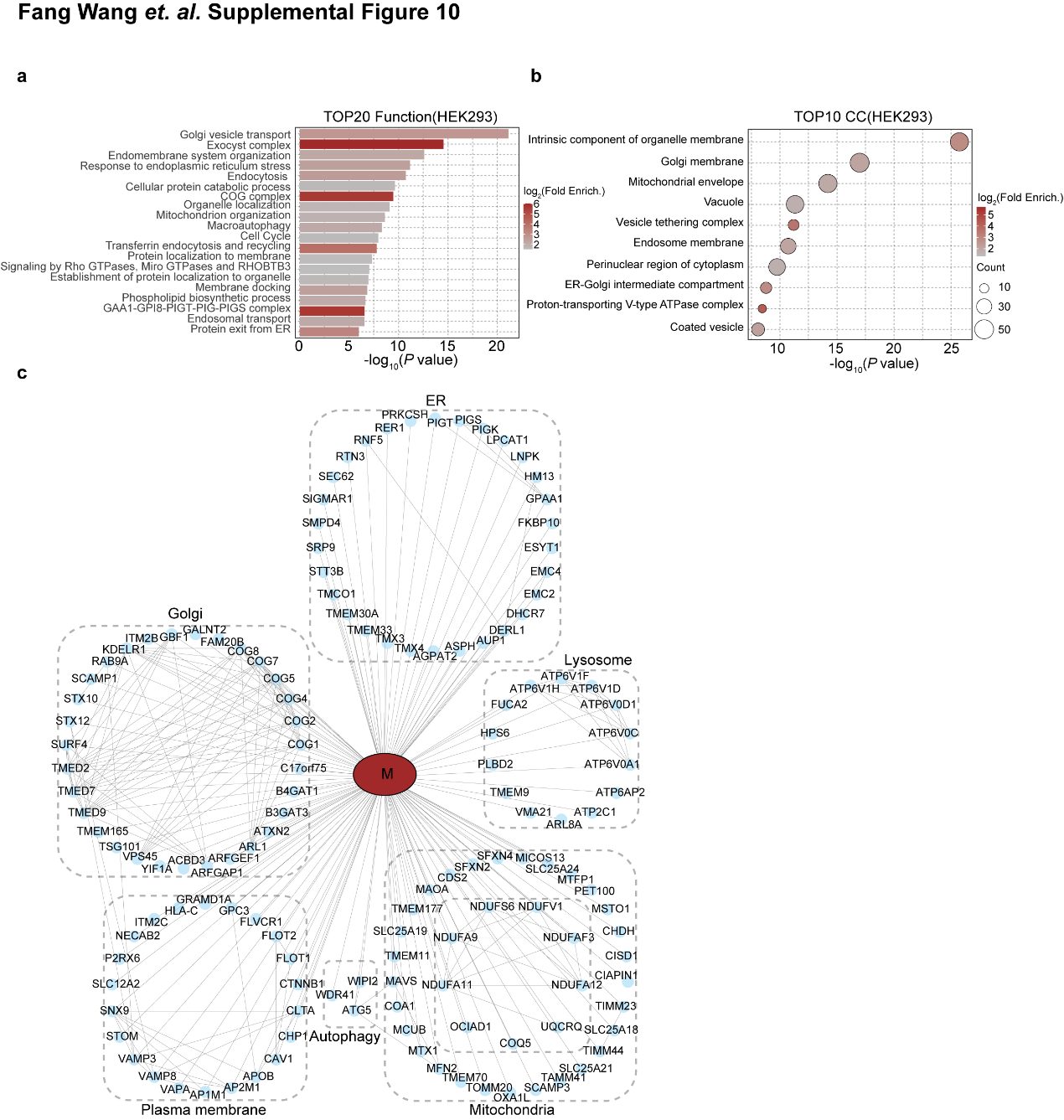


**Supplemental Figure 10. SARS-CoV-2 M interactome in HEK293 cells.**

**a, b** GO pathway analysis of SARS-CoV-2 M interactome in HEK293 cells. Proteins enriched > 20-fold comparing to control immunoprecipitation were included. The top 20 enriched GO biological functions (**a**) and top 10 enriched GO cellular components (CC) are shown (**b**). Circle sizes scale to number of detected proteins.

**c** Interactions between M (red ellipse) and human proteins (light-blue circles). Physical interactions among host proteins (thin black lines) were curated from STRING and visualized with Cytoscape. Results from 3 biologically independent samples were used for the study.


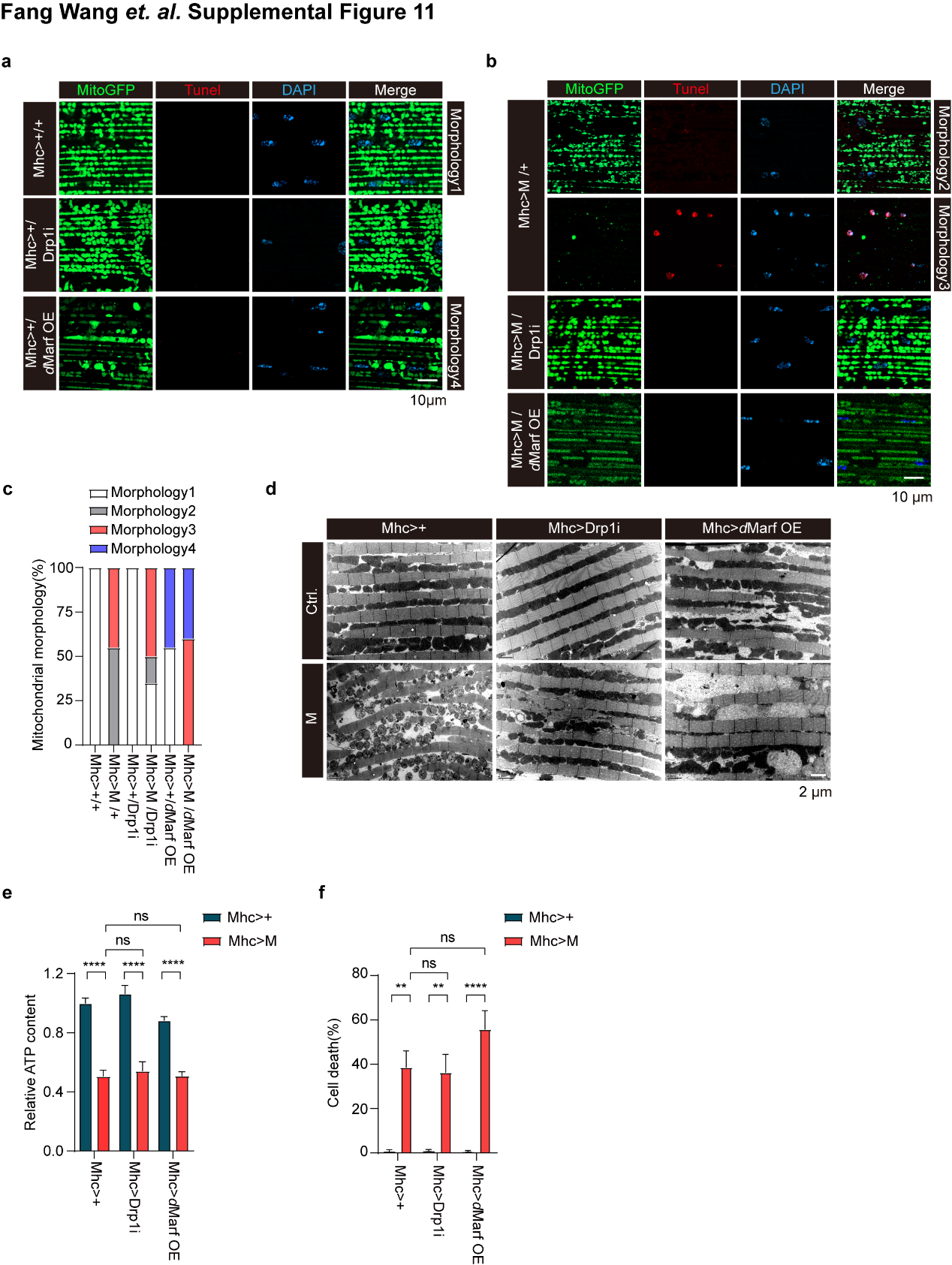


**Supplemental Figure 11. Inhibition of mitochondrial fragmentation unlikely restores mitochondrial function impaired by M.**

**a, b** Representative mitochondrial images of indirect flight muscle sections. The mitochondrial morphology was classified as four types, including Morphology 1 to 4 (**a**, **b**). Green: Mito-GFP; Red: Tunel staining; Blue: DAPI staining. Bar = 10 μm. Mhc>+/+: expression of mhc-gal4 alone. Mhc>+/Drp1i: Drp1 RNAi driven by mhc-gal4. Mhc>+/dMarf OE: *d*Marf expression driven by mhc-gal4. Mhc>M/+: M expression driven by mhc-gal4. Mhc>M/Drp1i: Expression of M and Drp1 RNAi driven by mhc-gal4. Mhc>M/*d*Marf OE: Expression of M and *d*Marf driven by mhc-gal4.

**c** Quantitative analysis of four types of mitochondrial morphology. Morphology 1-4 are quantified in fly indirect flight muscles with indicated genotype.

**d** Representative TEM images of fly indirect flight muscles with indicated genotypes. TEM images of male flies expressing mhc-gal4 driven SARS-CoV-2 M (M) followed by expressing either mhc-gal4 (Mhc>+) alone, mhc-gal4 driven Drp1 RNAi (Mhc>Drp1i), or mhc-gal4 driven expression of *Drosophila* Marf (Mhc>*d*Marf OE) were shown. Flies without M expression are included as control (Ctrl.). Bar = 2 μm.

**e** ATP contents of thorax muscle tissues from the indicated genotypes were measured and normalized against the protein levels. *****p<0.0001*, *ns:* no significance. Results from three independent experiments are expressed as mean ± SEM. The statistical analysis: Multi-way ANOVA followed by Dunnett’s test.

**f** Cell death analysis in flies with indicated genotypes. Quantification of M -induced cell death in the presence of Drp1 RNAi or *d*Marf overexpression. ***p <* 0.01, *****p <* 0.0001, *ns:* no significance*.* n > 10 flies/group. Results are expressed as mean ± SEM. The statistical analysis: Multi-way ANOVA followed by Dunnett’s test.


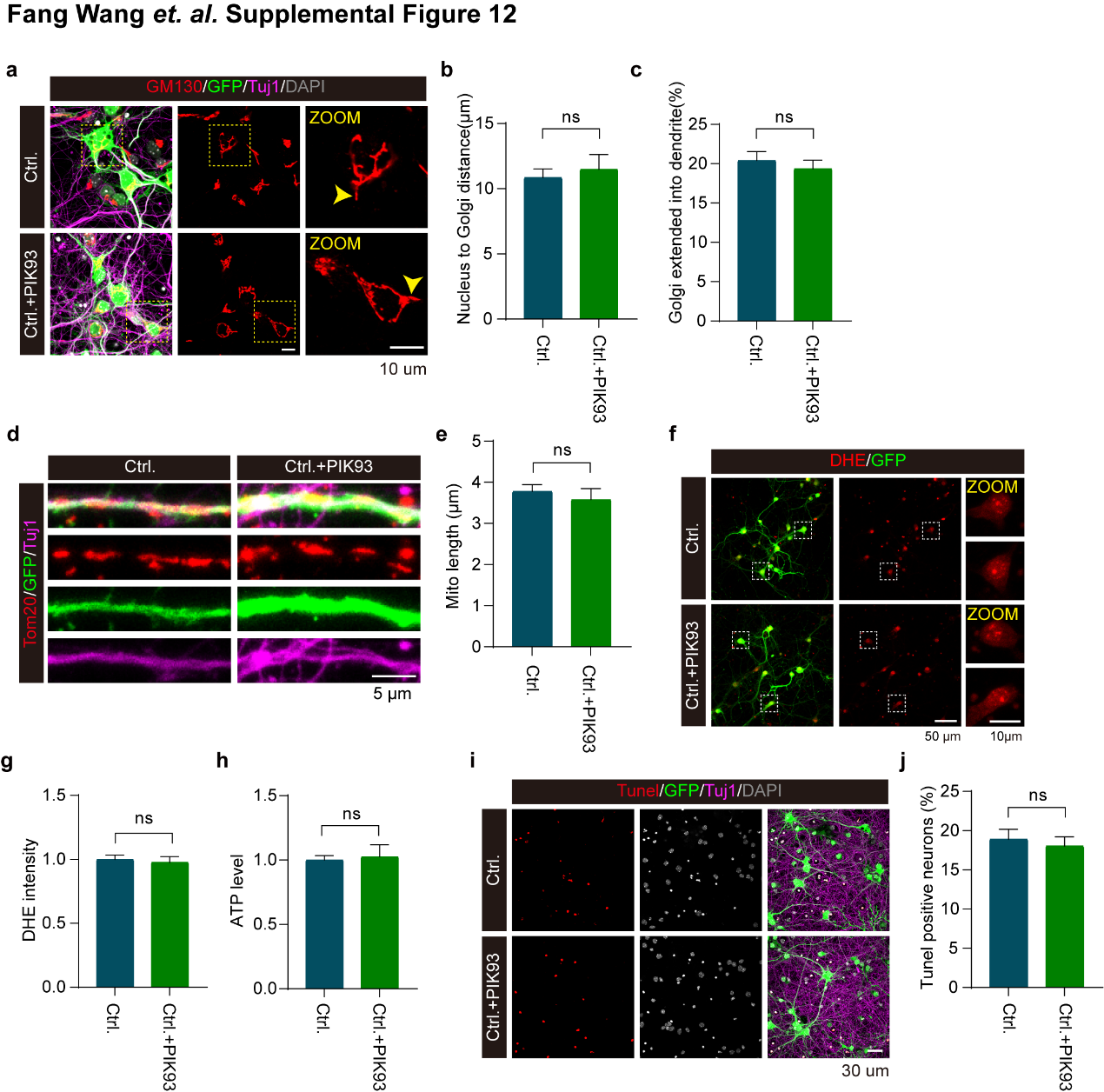


**Supplemental Figure 12. PI4KIIIβ inhibitor PIK-93 treatment has little impact on normal control neurons.**

**a-c** Inhibition of PI4KIIIβ does not affect the Golgi apparatus morphology in neurons. The primary cortical neurons were treated with either PIK93 (Ctrl+PIK93) or control solvent (Ctrl). The representative immuno-staining images are shown (**a**). Red: GM130 to label Golgi; Green: GFP to show AAV infection; Purple: Tuj1 to mark neuron; Grey: DAPI to label nuclei. Bar = 10 μm. A magnified image from gated frame of middle panel is shown (right panels, ZOOM). Yellow arrows indicate abnormal Golgi morphology. The distances from nucleus to Golgi (**b**) and the percentage of Golgi within dendrites (**c**) were quantified and analyzed. n > 50 neurons/group. ns = no significance. Results from three independent experiments are expressed as mean ± SEM. The statistical analysis: unpaired Student's t-test.

**d, e** Inhibition of PI4KIIIβ does not impact on neuronal mitochondrial morphology. The primary cortical neurons were treated with either PIK93 (Ctrl+PIK93) or control solvent (Ctrl). The representative immunofluorescent staining images are shown (**d**). Red: Tom20 to label mitochondria; Green: GFP to show AAV infection; Purple: Tuj1 to mark neuron. Bar = 5 μm. The mitochondrial length within dendrite were measured and analyzed (**e**). 3-5 secondary dendrites per neuron and > 50 neurons per indicated group were analyzed. ns = no significance. Results from three independent experiments are expressed as mean ± SEM. The statistical analysis: unpaired Student's t-test.

**f, g** Inhibition of PI4KIIIβ has little effect on ROS production in neurons. The primary cortical neurons were treated with either PIK93 (Ctrl+PIK93) or control solvent (Ctrl). DHE staining were performed. The representative images were shown (**f**). Red: DHE dye staining to label neuronal ROS; Green: GFP to show AAV infection. Bar = 50 μm. Enlarged images are shown (right panels, ZOOM), Bar = 10 μm. Neuronal relative DHE intensity were analyzed. n > 100 neurons/group. ns = no significance. Results from three independent experiments are expressed as mean ± SEM. The statistical analysis: unpaired Student's t-test.

**h** Inhibition of PI4KIIIβ does not significantly affect ATP production in neurons. The ATP contents are detected in neurons treated with (Ctrl+PIK93) or without PIK93 (Ctrl). The results are normalized to protein content. ns = no significance. Results from three independent experiments are expressed as mean ± SEM. The statistical analysis: unpaired Student's t-test.

**i, j** Inhibition of PI4KIIIβ does not induce neuronal apoptosis. The primary cortical neurons were treated with either PIK93 (Ctrl+PIK93) or control solvent (Ctrl). Immunofluorescent staining and Tunel staining were performed. Representative images were shown (**i**). Red: Tunel staining to label apoptosis; Green: GFP staining to show AAV infection; Grey: DAPI staining to label nuclei; Purple: Tuj1 to label neuron. Bar = 30 μm. The neuronal apoptosis (%) were analyzed. n > 100 neurons/group. ns = no significance. Results from three independent experiments are expressed as mean ± SEM. The statistical analysis: unpaired Student's t-test.
