# Supplementary material for: SARS-CoV-2 membrane protein induces neurodegeneration via affecting Golgi-mitochondria interaction
Source: Transl Neurodegener. 2024 Dec 27;13:68. doi: 10.1186/s40035-024-00458-1 (PMC11674522; doi:10.1186/s40035-024-00458-1)
Supplement: Supplementary file 2 — Additional file 2. Table S1. Antibody resource [file 40035_2024_458_MOESM2_ESM.docx]

**Table S1. Antibody Resource**

| **Reagent or Resource** | **SOURCE** | **IDENTIFIER** |
| --- | --- | --- |
| **Antibodies:** | | |
| DYKDDDDK Tag (D6W5B) Rabbit mAb | Cell Signaling Technology | 14793S |
| DYKDDDDK Tag (9A3) Mouse mAb | Cell Signaling Technology | 8146S |
| Anti GM130 antibody | Abcam | ab30637 |
| Anti Iba1 antibody | Wako | 019-19741 |
| Anti NeuN antibody | Cell Signaling Technology | 24307 |
| Anti MAP2 antibody | Cell Signaling Technology | 8707S |
| Anti Cleaved-Caspase3 antibody | Cell Signaling Technology | 9661S |
| Anti Bcl-2 antibody | Cell Signaling Technology | 15071 |
| Anti Tuj1 antibody | Cell Signaling Technology | 5568S |
| Anti GFAP antibody | Cell Signaling Technology | 12389 |
| Anti Aβ antibody | Cell Signaling Technology | 8243 |
| Anti GM130 antibody | Cell Signaling Technology | 12480 |
| Beta Actin Monoclonal antibody | Proteintech | 66009-1 |
| Anti BAX antibody | Proteintech | 50599-2 |
| Anti Tom20 antibody | Sata Cruz company | sc-17764 |
| Cy™2 AffiniPure Goat Anti-Mouse IgG (H+L) | Jackson ImmunoResearch | 115-225-166 |
| Cy™3 AffiniPure Goat Anti-Mouse IgG (H+L) | Jackson ImmunoResearch | 115-165-003 |
| Cy™2 AffiniPure Goat Anti-Rabbit IgG (H+L) | Jackson ImmunoResearch | 111-225-144 |
| Cy™3 AffiniPure Goat Anti-Rabbit IgG (H+L) | Jackson ImmunoResearch | 111-165-003 |
| Peroxidase AffiniPure Goat Anti-Mouse IgG (H+L) | Jackson ImmunoResearch | 115-035-003 |
| Peroxidase AffiniPure Goat Anti-Rabbit IgG (H+L) | Jackson ImmunoResearch | 111-035-144 |
| ANTI-FLAG® M2 Affinity Gel | Sigma-Aldrich | A2220 |
| Nanogold®-Fab' Goat anti-Rabbit IgG | Nanoprobes | 2004-1ML |
